# Age-dependent increase of oxidative stress regulates microRNA-29 family preserving cardiac health

**DOI:** 10.1038/s41598-017-16829-w

**Published:** 2017-12-04

**Authors:** Johanna Heid, Chiara Cencioni, Roberto Ripa, Mario Baumgart, Sandra Atlante, Giuseppina Milano, Alessandro Scopece, Carsten Kuenne, Stefan Guenther, Valerio Azzimato, Antonella Farsetti, Giacomo Rossi, Thomas Braun, Giulio Pompilio, Fabio Martelli, Andreas M. Zeiher, Alessandro Cellerino, Carlo Gaetano, Francesco Spallotta

**Affiliations:** 1 Division of Cardiovascular Epigenetics, Department of Cardiology, Goethe University, Frankfurt am Main, 60596 Germany; 2 National Research Council, Institute of Cell Biology and Neurobiology (IBCN), Rome, 00143 Italy; 3 Scuola Normale Superiore, Laboratory of Biology (Bio@SNS), c/o Istituto di Biofisica del CNR, Pisa, 56124 Italy; 4 Leibniz Institute on Aging - Fritz Lipmann Institute (FLI), Jena, 07745 Germany; 5 Vascular Biology and Regenerative Medicine Unit, Centro Cardiologico Monzino-IRCCS, Milan, 20128 Italy; 6 Laboratory of Cardiovascular Research, Department of Surgery and Anesthesiology, University Hospital Lausanne, Lausanne, 1015 Switzerland; 7 ECCPS Bioinformatics and deep sequencing platform, Max Planck Institute for Heart and Lung Research, Bad Nauheim, 61231 Germany; 8 Integrated Cardio Metabolic Centre, Department of Medicine, Karolinska Institutet, Huddinge, 141 57 Sweden; 9 Department of Veterinary Sciences, Faculty of Veterinary Medicine, University of Camerino, Camerino, (MC) 62032 Italy; 10 Dipartimento di Scienze Cliniche e di Comunità, Università degli Studi di Milano, Milan, 20122 Italy; 11 IRCCS-Policlinico San Donato, Moleculary Cardiology Laboratory, San Donato Milanese, (MI) 20097 Italy; 12 Internal Medicine Clinic III, Department of Cardiology, Goethe University, Frankfurt am Main, 60596 Germany

## Abstract

The short-lived turquoise killifish Nothobranchius furzeri (Nfu) is a valid model for aging studies. Here, we investigated its age-associated cardiac function. We observed oxidative stress accumulation and an engagement of microRNAs (miRNAs) in the aging heart. MiRNA-sequencing of 5 week (young), 12–21 week (adult) and 28–40 week (old) Nfu hearts revealed 23 up-regulated and 18 down-regulated miRNAs with age. MiR-29 family turned out as one of the most up-regulated miRNAs during aging. MiR-29 family increase induces a decrease of known targets like collagens and DNA methyl transferases (DNMTs) paralleled by 5´methyl-cytosine (5mC) level decrease. To further investigate miR-29 family role in the fish heart we generated a transgenic zebrafish model where miR-29 was knocked-down. In this model we found significant morphological and functional cardiac alterations and an impairment of oxygen dependent pathways by transcriptome analysis leading to hypoxic marker up-regulation. To get insights the possible hypoxic regulation of miR-29 family, we exposed human cardiac fibroblasts to 1% O_2_ levels. In hypoxic condition we found miR-29 down-modulation responsible for the accumulation of collagens and 5mC. Overall, our data suggest that miR-29 family up-regulation might represent an endogenous mechanism aimed at ameliorating the age-dependent cardiac damage leading to hypertrophy and fibrosis.

## Introduction

In western countries, the improvement of life style and accessibility to therapies significantly increased life expectancy to more than 75 years^1^ with relevant impact on the cost of health care^2,3^. Aging is characterized by a gradual physiological decline including a deficiency of antioxidant mechanisms causing a physiologic cell redox balance alteration^4,5^. The incessant increase of oxidative stress with age, associated to functional decline of cellular repair mechanisms, contributes to the onset and progression of typical age-associated pathologies, such as cardiovascular diseases^6–9^. Moreover, the consequent accumulation of reactive oxygen species (ROS) induces transforming growth factor beta (TGF-β) often activating fibroblasts and collagen deposition, typical initial steps of fibrotic diseases^10–12^.

The understanding of molecular pathways leading to aging and age-associated diseases will allow the development of new strategies in the direction of the so-called “healthy aging”^13^. In the past, the major obstacle to these studies was the lack of an appropriate vertebrate model with a lifespan compatible with laboratory research. Recently, the turquoise killifish *Nothobranchius furzeri* (Nfu), thanks to its relatively short life span (3–9 months) in comparison to *Danio rerio* (3–5 years) and *Mus musculus* (2 years), has been proposed as a new animal model for aging research in vertebrates^14,15^ especially after completing its genomic sequencing^16,17^. Remarkably, at the age of 5 weeks Nfu already reaches maturation and, within few additional months, some aging hallmarks including telomere shortening^18^, spinal curvature^19^, loss of mitochondrial function^20^ and neoplasia formation^21^ appear well detectable. Specifically, studies on aging brain performed in Nfu pointed out patterns involved in reduction of learning performances, gliosis and adult neurogenesis similar to those observed in aged humans, further supporting this model as valid in studies on aging^22^. Of note, these signs resemble some present in mammalians, and its exceptionally short lifespan put the Nfu in the spotlight as an interesting model for aging studies^23,24^. Nevertheless, the turquoise killifish remains a largely uncharacterized *in vivo* system in consequence of the limited number of studies performed so far. This poor knowledge might also limit the extent of the possible mechanistic investigations. The complete genome has been published just about two years ago^16,17^ and only very recently methodological details for genetic manipulation have been published but are not well established yet^25–28^. Therefore more investigations are needed to fully exploit the potential of this new *in vivo* model system.

MicroRNAs (miRNAs), short non-coding RNAs (ncRNAs) of about 20–22 nucleotides with gene expression regulatory functions, are involved in several molecular pathways responsible for lifespan regulation and in the onset of age-associated alterations^29–33^. At present, in Nfu about 165 conserved miRNAs have been associated with aging brain. Specifically, tumor suppressor miRNAs, such as miR-23a, miR-26a/b, miR-29a/b and miR-101a, were found upregulated, whereas oncogenic miRNAs, like miR-7a and members of the miR cluster 17∼92, were downregulated^34^. Despite age-dependent upregulation, antagonism of miR-29 exacerbates brain aging indicating that miR-29 has a protective role in neurons^35^. At present, no information is available about the expression and function of relevant miRNAs during Nfu aging in other important organs like the heart. Although, miRNA involvement in cardiac diseases has been extensively analyzed, the evaluation of hypertrophy or fibrosis, two signs of cardiac aging, has not been yet investigated in Nfu heart. MiRNAs play active roles during the pathophysiology of cardiovascular and cardiometabolic disorders putting them in evidence as promising biomarkers, both at diagnostic and prognostic level, as well as for novel potential therapeutic options^29,36–41^. Among the several examples related to their contributive role to cardiovascular disorders^29,36–41^ miR-34a, a miRNA sensitive to cardiac aging, has been described to regulate response to ischemia reperfusion injury after myocardial infarction^29^. Other miRNAs revealed an interesting potential as early predictive biomarkers being present in the systemic circulation. MiR-1, -133a, -133b, and -499-5p, in fact, increased within few hours after onset of symptoms associated with myocardial infarction^36^; miR-126 has been proposed as a reliable biomarker for endothelial senescence in elderly subjects and for aged diabetic patients^41^; miR-122, sensitive to hyperlipidemia, seems to correlate with severity of coronary artery disease^37^. Other miRNAs showed therapeutic potential and some promising results have been already obtained^38,39,42^ although their properties are still under intense investigation^39,40^. For example, miR-33a inhibition affects atherosclerosis progression^39^; and miR-181^38^ and miR-22^42^ showed ability to counteract hypertension. Moreover, miRNA pleiotropic regulatory activities and multi-targeting function shed light on their potential to limit restenosis after surgical procedures following occlusive vascular lesions^40^. In this perspective, miR-29 family members are of particular interest for cardiac pathophysiology. Myocardial infarction, in fact, induces a down-regulation of miR-29 which event, in turn, is partially responsible for fibrosis^43^. Interestingly, these miRNAs control the expression of collagen genes and are themselves controlled by TGF-β^44^ suggesting for a direct link between miR-29 family and the progress of inflammatory responses.

In this study, we investigated the cardiac phenotype of 5 week (young), 12–21 week (adult) and 28–40 week (old) fish heart, their miRNAome and the functional relevance of miR-29 family members in the cardiac aging process of Nfu.

## Results

### Oxidative stress accumulates in the aging heart of *Nothobranchius furzeri*

**Figure 1:**
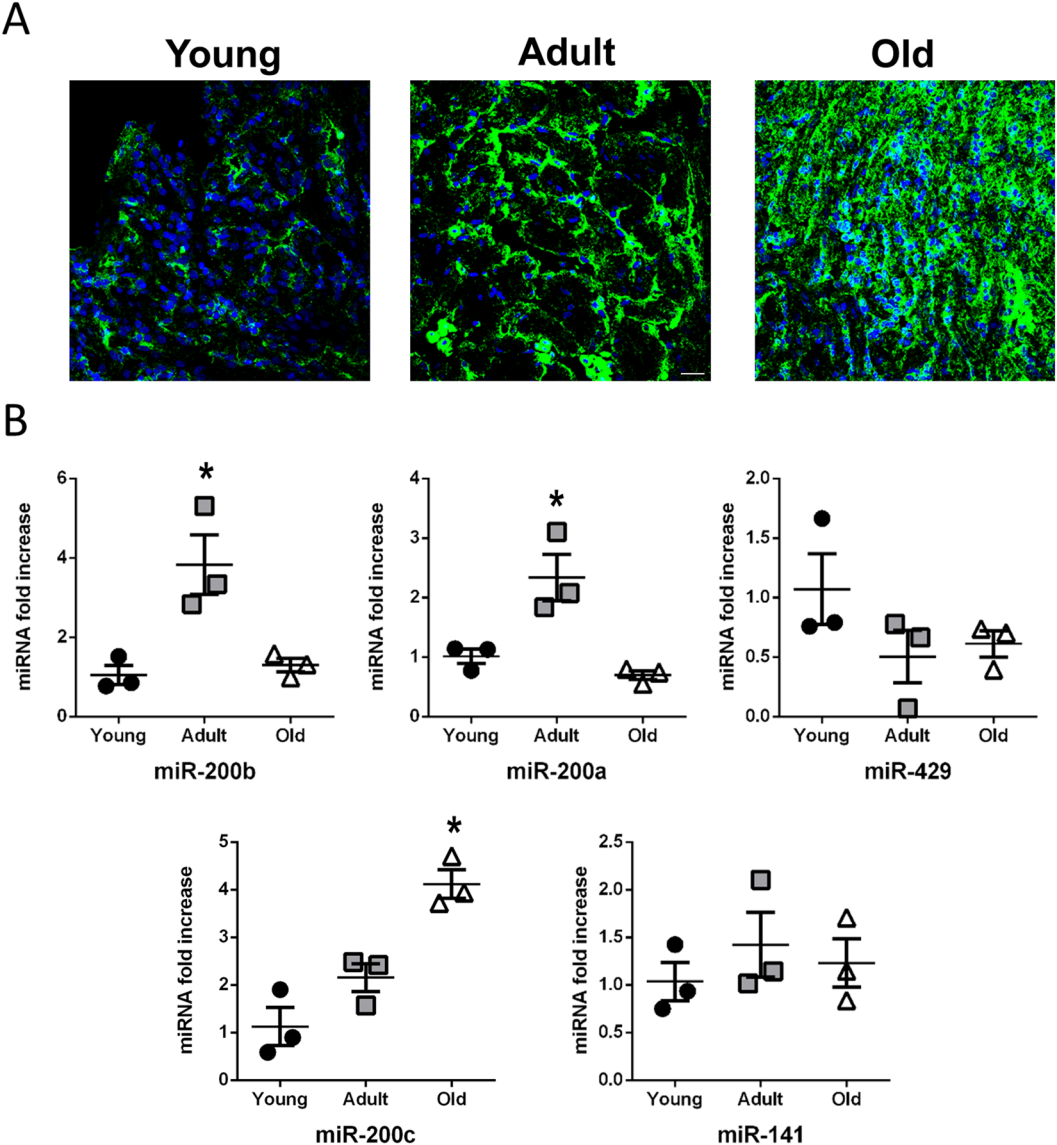
Oxidative stress accumulates in the aging heart of *N. furzeri*. (**A**) Representative confocal microscopy images of nitrotyrosine staining (green) in young (left panel), adult (middle panel) and old (right panel) Nfu hearts. Nuclei were counterstained with DAPI (blue). Calibration bar = 15 µm. (**B**) qRT-PCR analysis of miR-200 family members in young (black circles), adult (gray squares) and old (white triangle) Nfu hearts expressed as fold increase versus young samples (n = 3 at each age). *p < 0.05 Vs young.

Currently, little is known regarding Nfu heart as well as its morphology and function during aging. Post-mortem analyses of aged animals reported into the heart the presence of lesions, such as hypertrophy, dilatation and focal areas of phlogosis^21^. In an attempt to further characterize the heart of this new aging model, we analyzed oxidative stress levels in cardiac sections derived from young, adult and old Nfus. The oxidative stress damage was determined both by confocal analysis of total nitrotyrosinated proteins^45^ and by evaluation of miR-200 family member expression levels^46^, both increasing when ROS accumulates. Figure 1, panels A, B showed a progressive accumulation of nitrotyrosine staining and miR-200c levels during the aging process of Nfu.

### Aging regulates cardiac miRNAs expression in *Nothobranchius furzeri*

**Figure 2:**
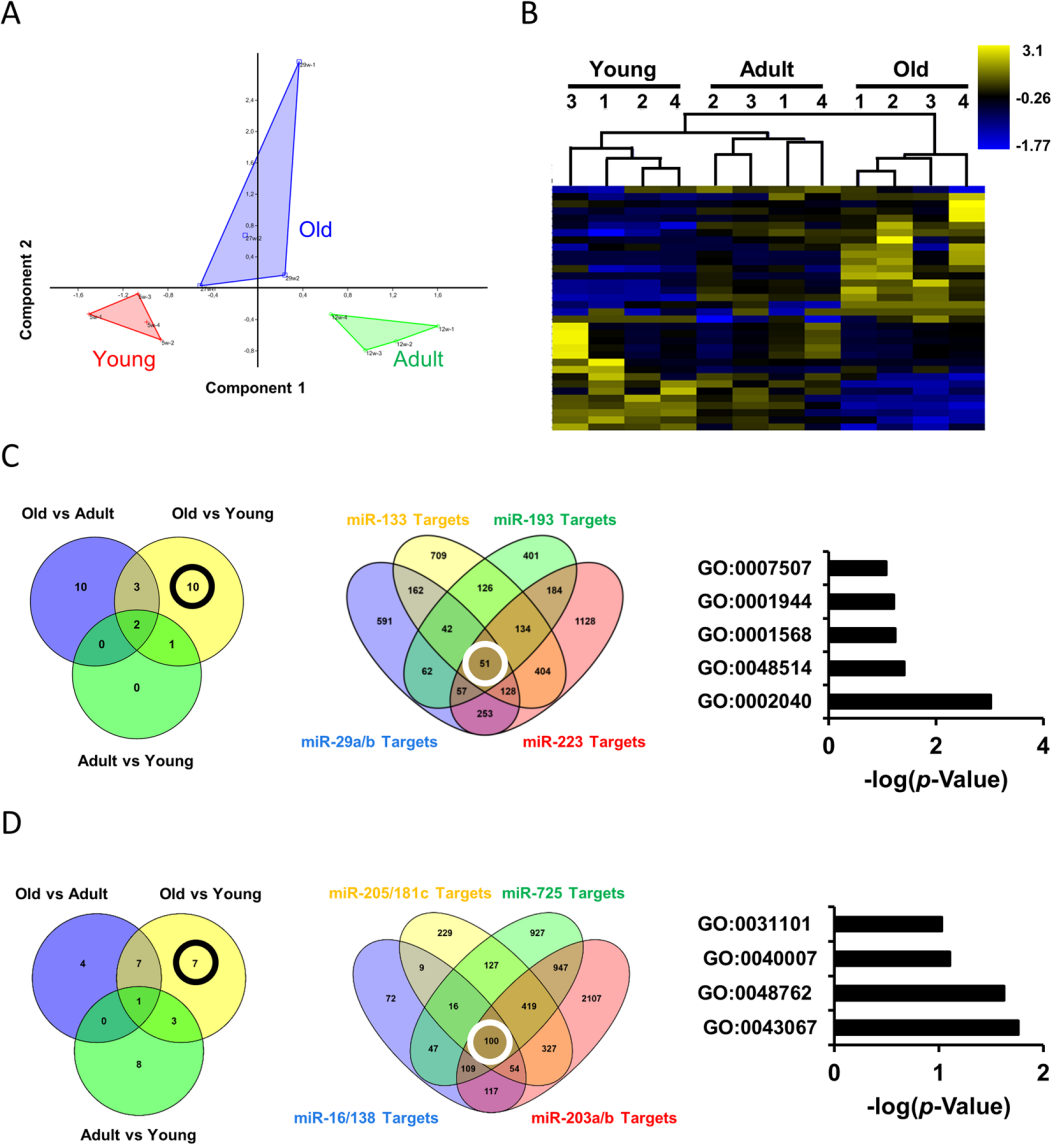
Aging regulates specific miRNAs in the heart of *N. furzeri*. (**A**) Principal Component Analysis (PCA) of miRNAs in young (5 weeks; red triangle), adult (12 weeks; green triangle) and old (27–29 weeks; blue triangle) Nfu hearts (n = 4 at each age). (**B**) Hierarchical clustering of miRNAs regulated more than ± 1.3 log2 fold change. Yellow and blue represent under- and over-expressed genes, respectively. (n = 4 at each age). (**C**) Venn diagrams depicting the distribution of up-regulated (left panel) miRNAs among young, adult or old Nfu hearts. Venn diagrams depicting the distribution of miR-133, miR-193, miR-29a/b, miR-223 predicted targets (middle panel). Gene ontology analysis of 51 common predicted targets (right panel). GO:0002040 sprouting angiogenesis; GO:0048514 blood vessel morphogenesis; GO:0001568 blood vessel development; GO:0001944 vasculature development; GO:0007507 heart development (**D**) Venn diagrams depicting the distribution of down-regulated (left panel) miRNAs among young, adult or old Nfu hearts. Venn diagrams depicting the distribution of miR-205/181c, miR-725, miR16/138, miR-203a/b predicted targets (middle panel). Gene ontology analysis of 100 common predicted targets (right panel). GO:0043067 regulation of programmed cell death, GO:0048762 mesenchymal cell differentiation; GO:0040007 growth; GO:0031101 fin regeneration.

The accumulation of oxidative stress and miR-200 family modulation prompted us to characterize the age-dependent regulation of the cardiac miRNome in Nfu. Prior work demonstrated the aging effect on miRNA expression specifically in the Nfu brain^34^. In the present work, we performed a complete miRNAome sequencing of the whole heart in three different age groups of Nfus: young (5 weeks; n = 4), adult (12 weeks; n = 4) and old (29 weeks; n = 4). Principal Component Analysis (PCA) was used to reduce the dimensionality of the dataset to visualize the global effect of age on the miRNome in a 2D plot (Fig. 2 panel A). The three groups were nicely separated according to age as indicated by the different colors: red (Young), green (Adult) and blue (Old). This complete separation was confirmed by unsupervised hierarchical clustering depicted as heatmap in Fig. 2 panel B. The most regulated microRNAs encompassing about ± 1.3 fold change between young and old (16 for up regulated miRNAs, 18 for down regulated miRNAs) were shown. After pairwise comparison of Young/Adult, Young/Old, and Adult/Old, 3, 16, and 15 miRNAs were found differentially expressed respectively with a value higher than ± 1.3 log2 fold change (see suppl. Table 1). From these data, Venn diagrams were generated to cluster selected up- and down-regulated miRNAs (Fig. 2 panels C, D). By this analysis, we identified 23 up-regulated and 18 down-regulated miRNAs by aging (Fig. 2 panels C, D). According to this analysis, as shown in Fig. 2C and D, middle panel and suppl. Table 2, miR-29a, miR-29b, miR-133, miR-193 and miR-223 were selected among the 10 most up-regulated miRNAs associated to the aging heart^43,47–49^. Targetscan software^50^ predicted about 51 common targets for miR-29a, miR-29b, miR-133, miR-193 and miR-223 (Fig. 2C, middle panel). The related gene ontology analysis suggested for an involvement in cardiovascular development, blood vessel morphogenesis and angiogenesis (Fig. 2C, right panel). By the same approach, we analyzed the 7 down-modulated miRNAs in the oldest fish (Fig. 2D, left panel; suppl table 1). The combination of their predicted targets revealed about 100 common genes (Fig. 2D, middle panel) involved in fin regeneration, growth, mesenchymal cell differentiation and apoptosis (Fig. 2D, right panel) as suggested by gene ontology analysis.

### Age-dependent miR-29 family up-regulation correlates with regulation of collagen and methylation levels in *Nothobranchius furzeri* heart

**Figure 3:**
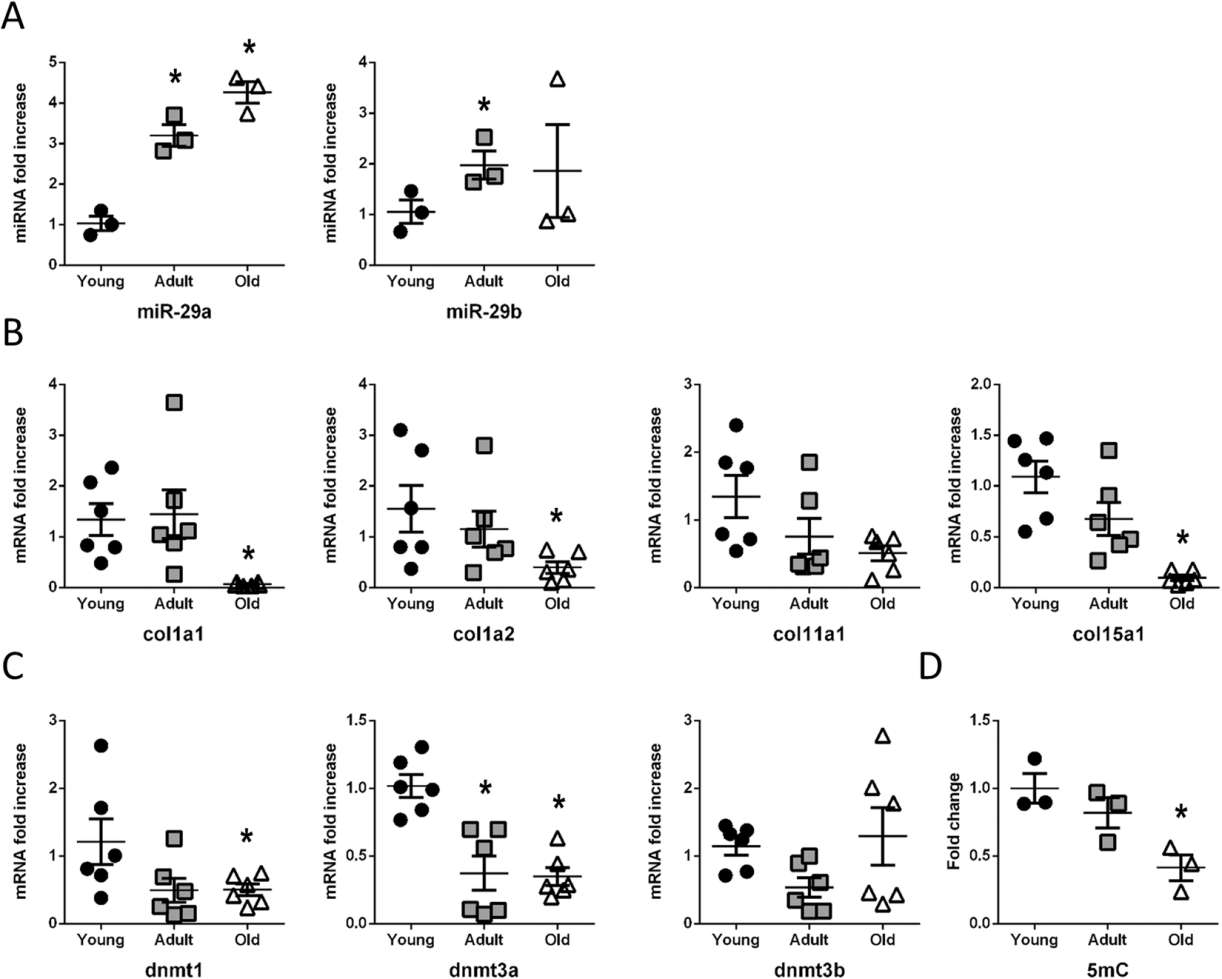
Age-dependent miR-29 family up-regulation affects collagen and methylation levels in *N. furzeri* heart. (**A**) qRT-PCR analysis of miR-29 family members in young (black circles), adult (gray squares) and old (white triangles) Nfu hearts expressed as fold increase versus young samples (n = 3 at each age). (**B**) qRT-PCR analysis of collagen mRNAs in young (black circles; n = 6), adult (gray squares; n = 6) and old (white triangles; n = 6) Nfu hearts expressed as fold increase versus young samples. (**C**) qRT-PCR analysis of DNA methyl transferases mRNAs (dnmts) in young (black circles; n = 6), adult (gray squares; n = 6) and old (white triangles; n = 6) Nfu hearts expressed as fold-change versus young samples. (**D**) Global DNA methylation quantification of 5-methyl cytosine (5mC) in young (black circles), adult (gray squares) and old (white triangles) Nfu hearts (n = 3 at each age). *p < 0.05 Vs young.

MiR-29 family, one of the most upregulated miRNAs, was further evaluated for its well-known role during aging and cardiovascular diseases. Specifically, members of the family have been found involved in the regulation of different cellular processes including gene expression, proliferation^51^, differentiation^52^, tumorigenesis^53^, apoptosis^54^, senescence^55^ and extracellular matrix deposition^56^. Moreover, miR-29 family play a role during DNA methylation / demethylation control^57^ and cellular reprogramming^58^. Specifically, in the heart, miR-29 family up-regulation is associated with cardiac development and growth regulation^59^ whereas its down-regulation is involved in cardiac tissue remodeling after myocardial infarction^43^. MiR-29a up-regulation was confirmed by specific qRT-PCR on the whole heart of old Nfu (Fig. 3 panel A). Then, the expression level of fibrotic markers, known target of the family, such as collagens, and of DNA methyltransferases (dnmts) was evaluated in the heart of three different stages of Nfu lifespan. Figure 3 panel B shows an age-dependent decrease of collagen 1A1 (col1a1), collagen 1A2 (col1a2), collagen 11A1 (col11a1) and collagen 15A1 (col15a1). Figure 3 panel C shows a cardiac transcript decrease levels of dnmt1 and dnmt3a (Fig. 3 panel C) paralleled by a global reduction of 5-methylcytosine (5mC) content (Fig. 3 panel D). All these evidences were also confirmed in human primary cardiac fibroblasts (HCF) isolated from the heart of fetal, adult and old donors. In HCF derived from old donors, all miR-29 family members were upregulated (Figure S1 panel A), with a parallel decrease of col3a1 expression (Figure S1 panel B) and global 5mC level (Figure S1 panel C).

### Oxidative stress induces expression of microRNA-29 family

**Figure 4:**
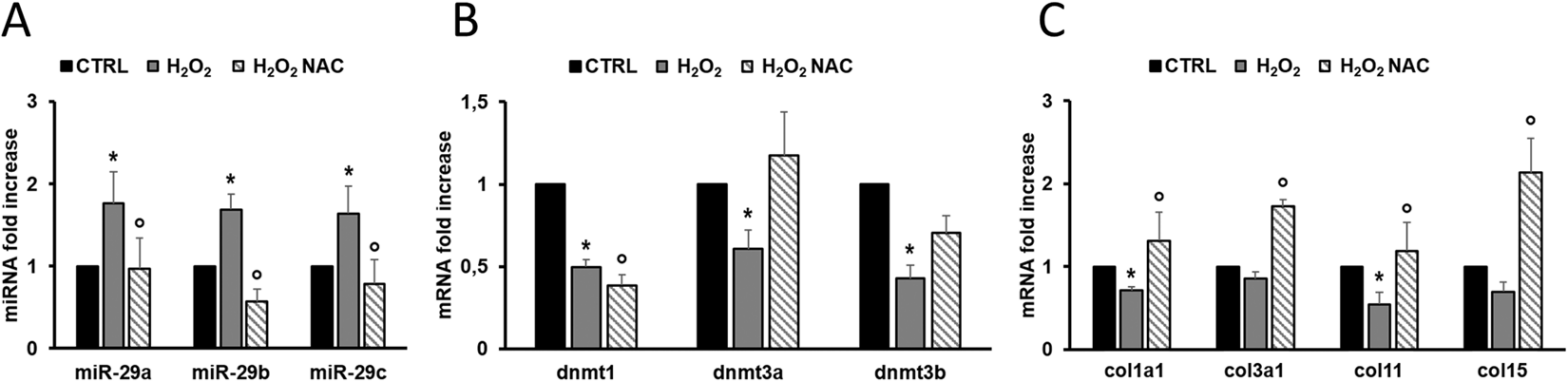
Oxidative stress affects miR-29 family and its targets. (**A**) qRT-PCR analysis of miR-29 family members in human cardiac fibroblasts (HCF) cultured in control conditions (CTRL; black bars; n = 7), in the presence of H_2_O_2_ (gray bars; n = 7) and of H_2_O_2_ + NAC (striped bars; n = 6) expressed as fold-change versus control samples. (**B**) qRT-PCR analysis of dnmt mRNAs in human cardiac fibroblasts (HCF) cultured in control conditions (CTRL; black bars; n = 8), in the presence of H_2_O_2_ (gray bars; n = 8) and of H_2_O_2_ + NAC (striped black bars; n = 4) expressed as fold-change versus control samples. (**C**) qRT-PCR analysis of collagen mRNAs in human cardiac fibroblasts (HCF) cultured in control conditions (CTRL; black bars; n = 8), in the presence of H_2_O_2_ (gray bars; n = 8) and of H_2_O_2_ + NAC (striped black bars; n = 4) expressed as fold-change versus control samples. *p < 0.05 Vs control; °p < 0.05 Vs H_2_O_2_.

To investigate the potential relationship between the accumulation of ROS in the heart (see Fig. 1 panels D and E) and the increase in miR-29 family member expression (Fig. 3 panel A), differentiated H9C2 rat cardiomyoblasts and human cardiac fibroblasts (HCF) were exposed to H_2_O_2_^46^. miR-29 family resulted sensitive to 200 µM H_2_O_2_ already after 24 h of treatment (Figure S2 panel A and Fig. 4 panel A). Noteworthy, the ROS scavenger N-acetyl cysteine (NAC) counteracted the effect of H_2_O_2_ on expression of miR-29 expression (Figure S2 panel A and Fig. 4 panel A) suggesting a link between miR-29 and oxidative stress. As HCF are the primary source of collagen in a damaged heart^60^, all further experiments, aiming at evaluating the contributive role of miR-29 family in cardiac fibrosis, were conducted taking advantage from the availability of these primary cells. Intriguingly, after treatment with H_2_O_2_, the transcripts coding for miR-29 target genes, including col1a1, col11 and dnmt1, dnmt3a and dnmt3b, were down regulated while NAC partially restored their normal mRNA levels (Fig. 4 panels B and C). Similar results were obtained in HCF treated for 24 h with 10 µM Carbonyl cyanide m-chlorophenyl hydrazone (CCCP), a chemical inhibitor of oxidative phosphorylation^61^ (Figure S2 panel B). Taken altogether, these data suggested that miR-29 family is regulated by ROS.

### miR-29 family knock-down changes global methylation level and collagen expression in Zebrafish

**Figure 5:**
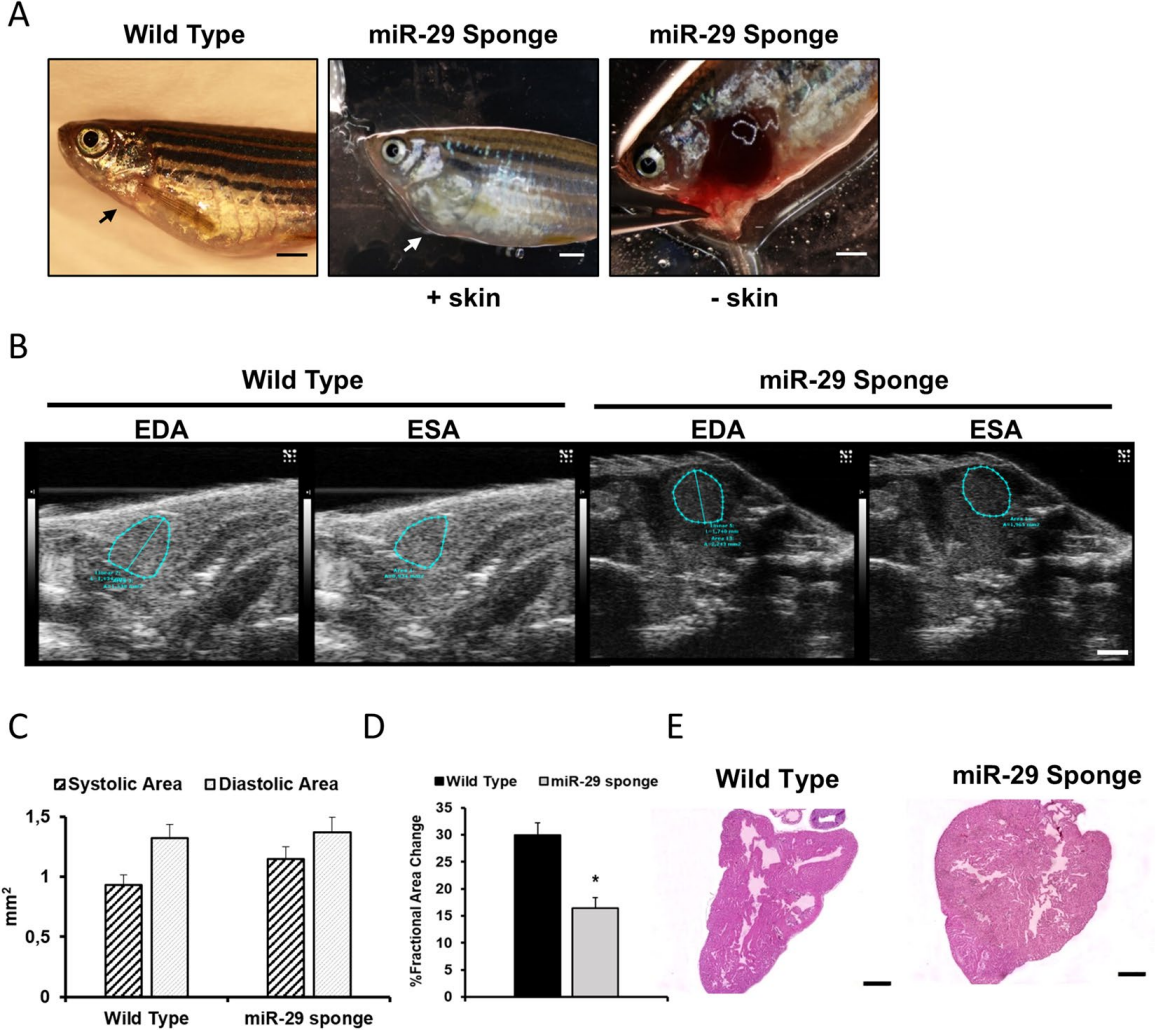
miR-29 family knock-down induces morphological and physiological changes in the heart of Zebrafish. (**A**) Representative images of Wild Type Zebrafish (left panel) and miR-29-sponge Zebrafish: view from outside (middle panel (+skin)) and with open chest (right panel (−skin)). Note the convexity in the cardiac region of miR-29 Sponge animal compared to Wild Type (black arrow) indicated by the white arrow. Calibration bar = 1 mm (**B**) Representative echocardiography of Wild Type (left panels) and miR-29-sponge (right panels) Zebrafish hearts showing end-diastolic area (EDA; first and third panel) and end-systolic area (ESA; second and fourth panel) Calibration bar = 1 mm. (**C**) Graph shows systolic area (black striped bars) and diastolic area (light gray bars) in Zebrafish hearts of Wild Type (n = 9) and miR-29-sponge Zebrafish (n = 13). (**D**) Graph shows Fractional Area Change (FAC) in Wild Type (black bar; n = 9) and miR-29-sponge (gray bar; n = 13) Zebrafish hearts. (**E**) Representative hematoxylin eosin staining in Wild Type (left panel) and miR-29-sponge (right panel) Zebrafish ventricles. Calibration bar = 100 µm. (**D**) *p < 0.05 Vs Wild Type.

**Figure 6:**
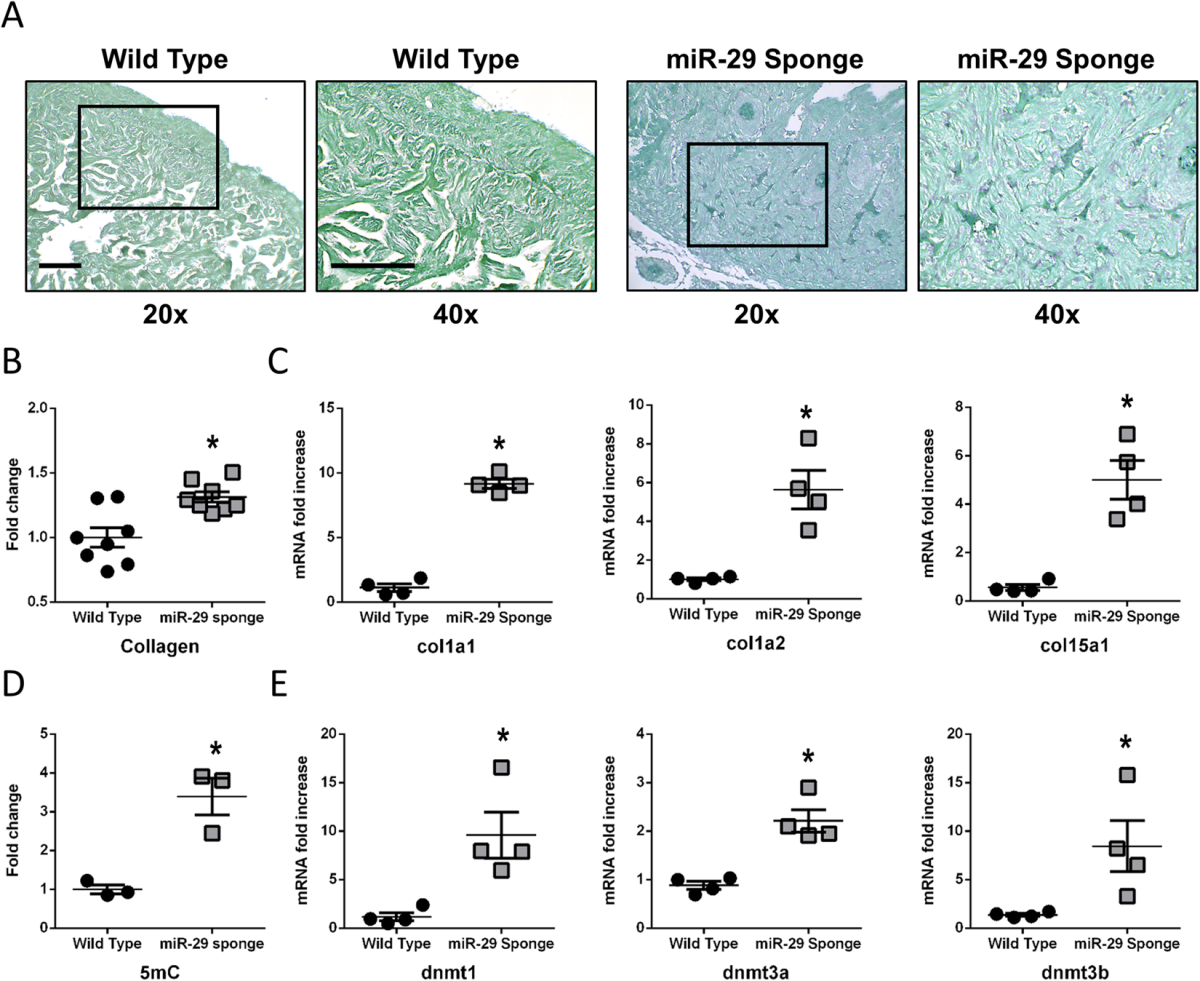
miR-29 family knock-down associates with collagen deposition and DNA methylation increase in the Zebrafish heart. (**A**) Representative Fast Green Sirius Red staining of Wild Type (left panels) and miR-29-sponge (right panels) Zebrafish ventricles. Collagenous proteins are depicted in light purple and non-collagenous proteins in green. Magnification: 20x in first and third panel and 40x in second and fourth panel. Calibration bar = 25 µm (**B**) Collagen deposition quantification in sections derived from Wild Type (black circles; n = 8) and miR-29-sponge (gray squares; n = 8) Zebrafish hearts. (**C**) qRT-PCR analysis of collagen mRNAs in Wild Type (black circles; n = 4) and miR-29-sponge (gray squares; n = 4) Zebrafish hearts expressed as fold increase versus Wild Type samples. (**D**) Global DNA methylation quantification of 5mC in Wild Type (black circles; n = 3) and miR-29-sponge (gray squares; n = 3) Zebrafish hearts expressed as fold-change versus Wild Type samples. (**E**) qRT-PCR analysis of dnmt mRNAs in Wild Type (black circles; n = 4) and miR-29-sponge (gray squares; n = 4) Zebrafish hearts expressed as fold increase versus Wild Type samples. *p < 0.05 Vs Wild Type.

To functionally analyze the role of miR-29 in the heart we generated a transgenic zebrafish model where miR-29 biological activity was antagonized by the stable expression of a competitive inhibitor (a 3′-UTR containing seven repeats of the miR-29 binding site: miR-29-sponge) under the control of the actin beta-2 promoter (actb2:eGFP-sponge-29) of *Danio renio* ^35,62^. Upon miR-29 knockdown, no significant changes were detected in terms of survival during the first year of age (data not shown). However, we observed the progressive formation of cardiac edema (Fig. 5 panel A). In order to further investigate the effect of miR-29 sponge on cardiac function we performed 2D-echo on wild type (wt) and miR-29-sponge fish (Fig. 5 panels B, C and D). We found a highly compromised fractional area change (FAC) in the sponge-29 animals without significant changes in end diastolic area (EDA) and end systolic area (ESA) (Fig. 5 panel D and C). Specifically, miR-29 sponge fish showed a FAC of 16%, whereas control animals had values of about 30% (Fig. 5 panel D). Morphologically, we found a significant cardiac spherization in fish injected with miR-29-sponges detectable by 2D-echo analysis (Fig. 5 panel B) and visible hypertrophy that were confirmed by histological examinations (Fig. 5 panel E). To further evaluate the effect of the miR-29-sponge, collagen deposition was evaluated (Fig. 6 panels A, B, C). Histological analysis revealed a significant amount of tissue positive to Fast Green/Sirius Red staining suggesting a global increase of collagen deposition in miR-29-sponge heart samples compared to controls (Fig. 6 panel A, B). The increased expression levels of col1a1, col1a2 and col15a1, all known direct targets of miR-29, further confirmed an accumulation of collagen deposition in the miR-29-sponge heart (Fig. 6 panel C). Interestingly, the accumulation of global 5-methyl cytosine (5mC) levels (Fig. 6 panel D) was paralleled by an increase in dnmt level (Fig. 6 panel E) in the same animals.

### Hypoxia induces hypermethylation and fibrosis by miR-29 family down-modulation

**Figure 7:**
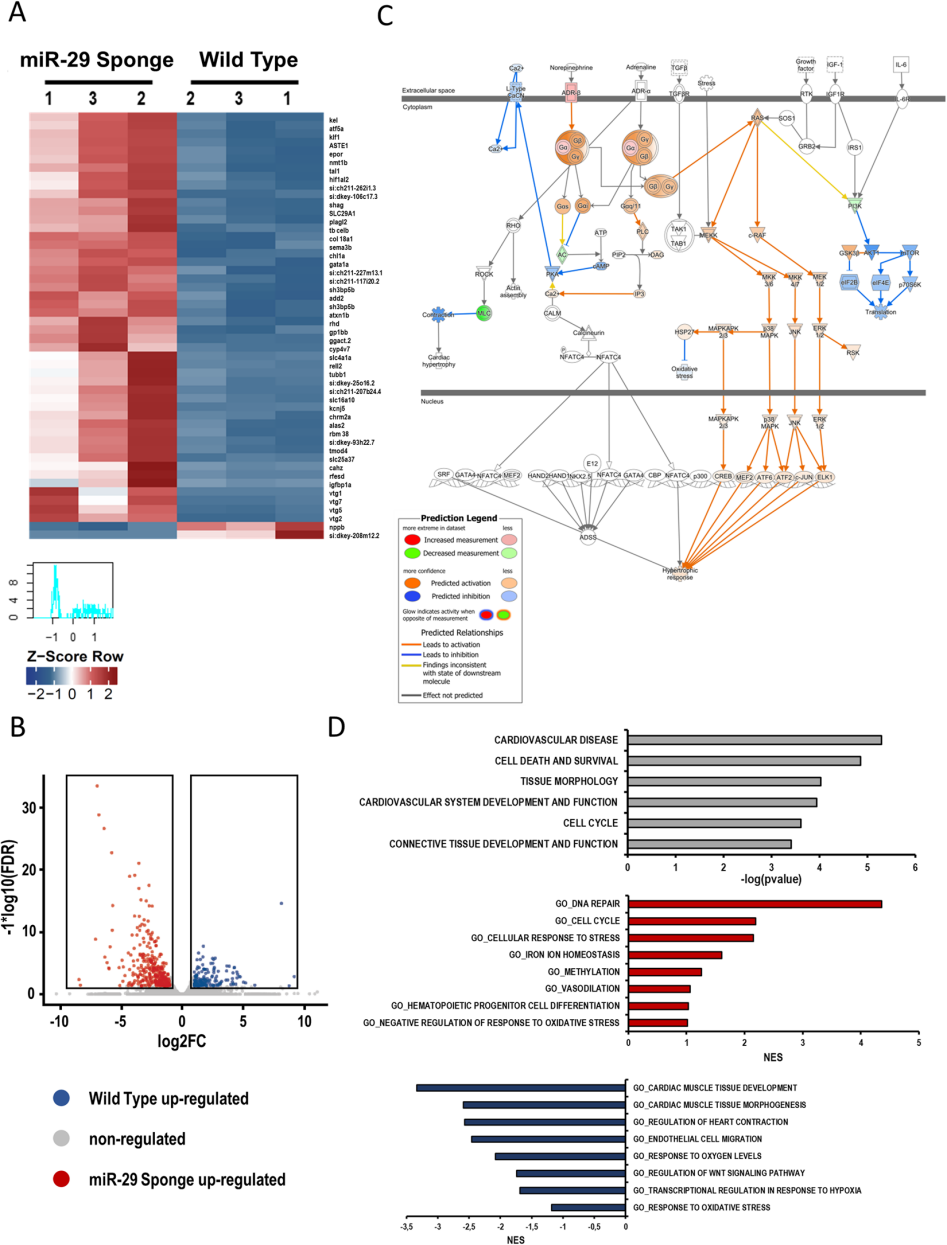
Identification of miR-29 associated cardiac transcriptome. (**A**) Heatmap showing the 50 most differentially regulated genes in the heart of Wild Type and miR-29-sponge Zebrafish identified by total RNA sequencing analysis. Red and blue represent over- and under-expressed genes, respectively. List of genes is provided also in supplemental table 5. (**B**) Volcano plot of differentially regulated genes expressed in the heart of Wild Type and miR-29-sponge Zebrafish. Red dots show miR-29-sponge up-regulated genes, blue dots show Wild Type up-regulated genes. (**C**) Canonical pathway analysis of Ingenuity Pathway Analysis prediction for hypertrophic response activation. Orange shapes represent activation; blue shapes represents inhibition. The intensity of color represents the degree of prediction. (**D**) Disease pathway analysis of modulated genes in miR-29-sponge Zebrafish (gray bars). Biological function gene ontology of up-regulated genes in miR-29-sponge Zebrafish hearts (red bars). Biological function gene ontology of genes up-regulated genes in Wild Type Zebrafish hearts (blue bars).

**Figure 8:**
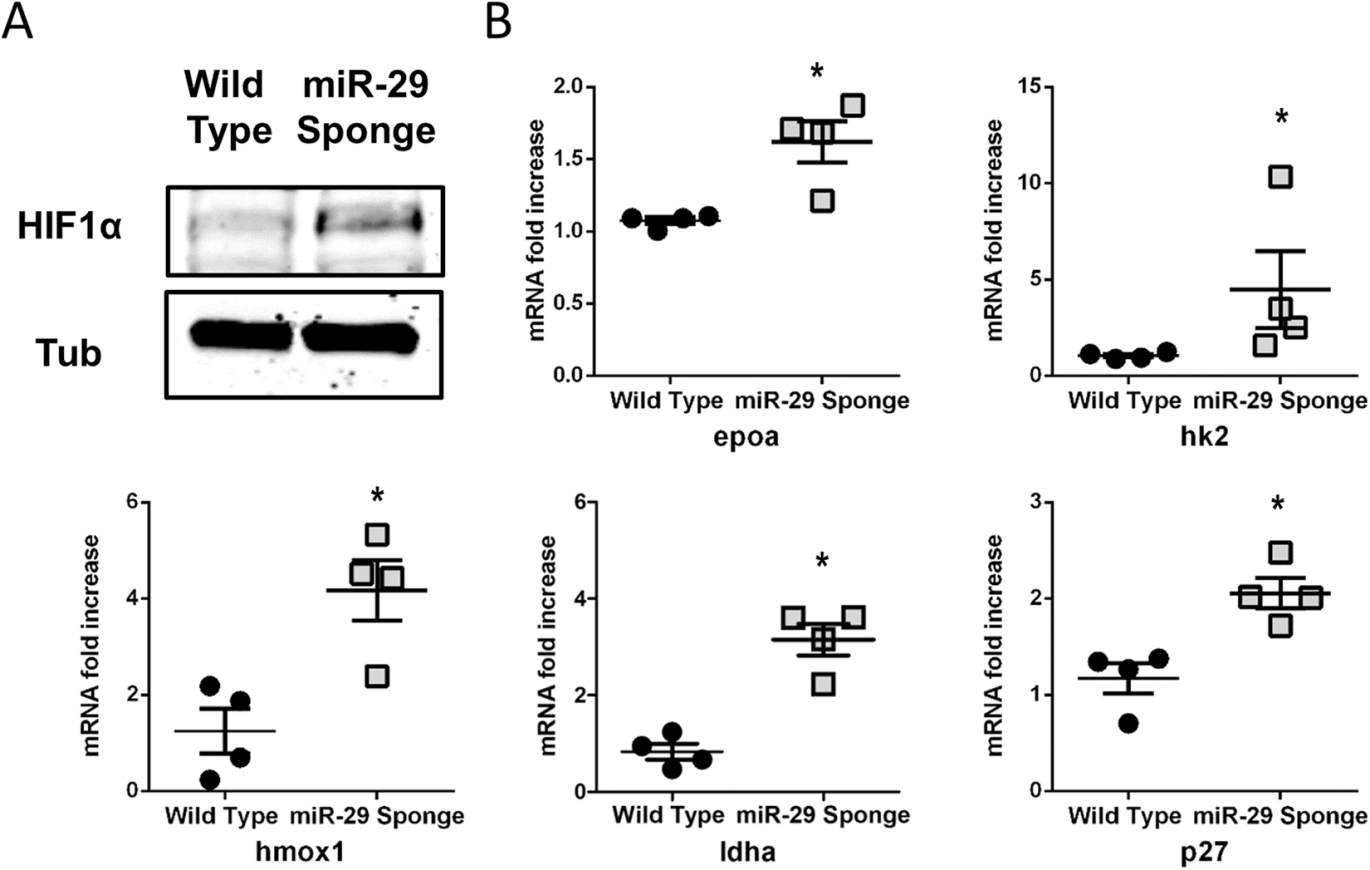
Hypoxic markers accumulate in miR-29-sponge Zebrafish hearts. (**A**) Representative western blot analysis of hypoxia inducible factor 1α (HIF1α) expression in Wild Type and miR-29-sponge Zebrafish heart. In each condition, α-tubulin was used as loading control. Three independent experiments were performed. Full-length blot is presented in Supplementary Figure 6.(**B**) qRT-PCR mRNA analysis of hypoxia associated genes: erythropoietin alpha (epoa); hexokinase2 (hk2); heme oxygenase1a (hmox1a); lactate dehydrogenase A (ldha); cyclin-dependent kinase inhibitor 1B (p27) in Wild Type (black circles; n = 4) and miR-29-sponge (gray squares; n = 4) Zebrafish hearts expressed as fold-change versus Wild Type samples. *p < 0.05 Vs Wild Type.

To further explore the effect of miR-29 family depletion on the cardiac molecular phenotype, RNA sequencing (RNA-seq) was performed on the whole heart of wt and miR-29-sponge fish. This analysis revealed that the two conditions were associated to clearly separated gene expression patterns (Fig. 7 panel A; suppl. table 3, 4, 5). Interestingly, 350 transcripts were found up regulated in the miR-29-sponge compare to Wild type hearts (suppl. table 3) whereas 162 transcripts were down regulated (Fig. 7 panel B; suppl. table 4). Among all these transcripts, a set of genes associated with heart, methylation, fibrosis and hypoxia were validated by specific qRT-PCR (Figure S3 panel A). Ingenuity pathway analysis on genes regulated by miR-29 depletion ( ± 0.5 log2 fold change, basemean > 5, fdr < 0.05) revealed a predicted activation of the hypertrophic response in miR-29 sponge fish, perfectly fitting with the observed cardiac phenotype (Fig. 7 panel C). All the transcription factors, namely creb, mef2c, atf6, c-jun and elk1, upstream of the predicted activation of hypertrophic response revealed by Ingenuity pathway analysis (Fig. 7 panel C) were also validated by single qRT-PCR (Figure S3 panel B). Cardiovascular disease, cell death, survival and tissue morphology were among the most affected disease pathway revealed by Ingenuity software (Fig. 7 panel D; gray bar graph). Moreover, gene ontology analysis of biological functions obtained by Gene set enrichment analysis (GSEA) pointed out an up-regulation of cellular response to stress and methylation (Fig. 7 panel D; red bar graph) as well as a down-modulation of response to oxidative stress and cardiac morphology and functions (Fig. 7 panel D; blue bar graph), which fully correlate with our experimental evidences on miR-29 sponge fish in comparison to Wild Type. The apparent impairment of the oxygen-dependent pathways prompted us to evaluate the presence of some hypoxic markers in the heart of miR-29-sponge fish. In these animals, we observed a stabilization of the hypoxia-inducible factor 1-alpha (HIF1alpha) protein (Fig. 8 panel A) and the upregulation of the hypoxic makers, like hexokinase 2 (hk2), lactate dehydrogenase A (ldha), heme oxygenase 1a (hmox1a), erythropoietin a (epoa) and p27 (Fig. 8 panel B).

**Figure 9:**
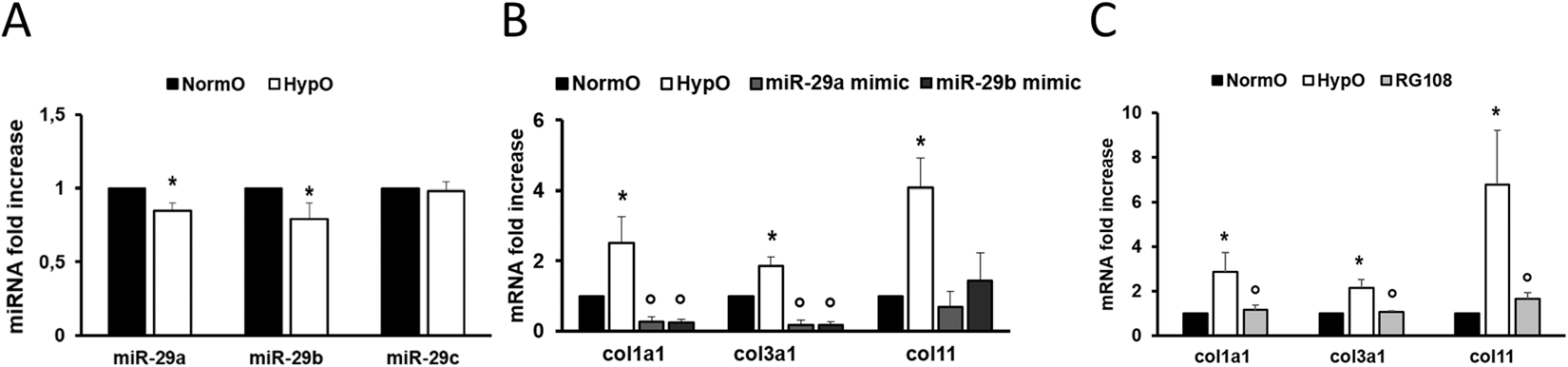
Hypoxia affects miR-29 family and its related targets. **(A**) qRT-PCR analysis of miR-29 family members in HCFs under normoxic conditions (NormO; black bars) and 48 h hypoxia (HypO; white bars; n = 7) expressed as fold-change versus normoxic samples. (**B**) qRT-PCR analysis of col mRNAs in HCFs under normoxic conditions (NormO; black bars; n = 6), under hypoxia prior (HypO; white bars; n = 6) and after transfection with miR-29a mimic (miR-29a; gray bar; n = 3) or miR-29b mimic (miR-29b; dark gray bar; n = 3) expressed as fold increase versus normoxic samples. (**C**) qRT-PCR analysis of col mRNAs in HCFs under normoxic conditions (NormO; black bars), under hypoxia in the absence (HypO; white bars) or presence of RG108 (RG108; gray bar) expressed as fold increase versus normoxic samples (n = 5). *p < 0.05 Vs normoxia; °p < 0.05 Vs hypoxia).

To investigate the effect of hypoxia on miR-29 family and its targets, HCFs were exposed to 1% O_2_ concentration for 48 hours. In all experiments the level of miR-210, a hypoxia sensitive miRNA^63,64^, and HIF-1α protein were evaluated as readouts for hypoxic condition (Figure S4 panels A and B)^65^. In this experimental setting miR-29a and b down-regulation was detected (Fig. 9 panel A). The sensitivity of miR-29a and miR-29b to O_2_ reduction was confirmed also by pri-miR-29 analysis. Specifically, supplemental figure S4 panel C shows a significant decrease of pri-miR-29a/b after hypoxia whereas the level of pri-miR-29b/c remained stable. Interestingly, we found a strong increase of collagen and dnmt expression levels under hypoxic condition (Fig. 9 panel B and S4 panel D) paralleled by an increased in DNMT activity (Figure S4 panel E) and a possible consequent accumulation of global 5mC content (Figure S4 panel F). Of note, exogenous expression of miR-29a/b mimics rescued the hypoxic and fibrotic phenotype (Fig. 9 panel B) suggesting a possible protective role of miR-29 family to counteract hypoxia-related collagen deposition and consequently fibrosis. Consistently, the DNMT inhibitor RG108 exerted the same effect of miR-29a or miR-29b mimics (Fig. 9 panel C), further suggesting a potential role for miR-29 family in preventing hypoxia-dependent hypermethylation via down-regulation of DNMTs^66,67^.

## Discussion

In the present study, we investigated changes in the global cardiac miRNA expression pattern and global DNA methylation during Nfu lifespan. During its relatively short life the turquoise killifish displays many of the classical morphological signs of aging including spinal curvature and neoplasia formation as well as molecular and cellular alterations like telomere shortening and reduction of mitochondrial function^18–21^. Remarkably, changes in cardiac function and molecular phenotype were unexplored, so far. To gain this evidence we characterized the cardiac phenotype of old Nfu, which prompted us to focus our attention on miR-29 family. To better evaluate the contributive role of miR-29 during cardiac aging, the *in vivo* genetic manipulation has been performed in the zebrafish model, a well-established organism especially suitable for genetic engineering although not specifically indicated for aging studies due to its relatively long lifespan. The validation of our *in vivo* evidences was realized in well-characterized cardiac cellular models of human and rat origin due to the lack of protocols for cardiac fibroblast isolation from Nfu. At present, in fact, only fibroblasts from Nfu skin have been successfully isolated^68^. On the other hand, the confirmation of our evidences in mammalian cells, supports Nfu as an aging model indicating the presence also in this system of mechanisms conserved across vertebrate classes.

Specifically, miRNA-seq analysis identified 5 up-regulated cardiac specific miRNAs (miR-29a, miR-29b, miR-133, miR-193 and miR-223) previously identified for being regulators of cardiac development and homeostasis (Fig. 2). In the cardiovascular system, miR-29 family has multiple roles: i) is known to be involved in atrial fibrillation^69^; ii) may act as a negative regulator of fibrosis counteracting miR-21 function^43,70,71^; iii) controls cardiomyocytes apoptosis and aortic aneurism formation^30,72^. In addition, miR-29 has been described to be up regulated during cardiac aging in mouse^30^. Because of its biological relevance in cardiac pathophysiology, we focused our attention on miR-29 family. Noteworthy, this miRNA family plays a fundamental role in the regulation of DNA methylation by direct targeting of virtually all main enzymes of the DNA methylation and demethylation machinery such as DNMTs, ten eleven translocation proteins (TET) and the Thymine DNA Glycosylase (TDG)^73^. Interestingly, the analysis of miRNA expression performed in different Nfu organs, including brain, liver, heart and skin, revealed miR-29 family together with miR-27d as one of the only two consistently up-regulated miRNAs during aging in all the four tissues (Figure S5)^34^.

Recently it has been showed that Nfu is a good model to investigate the role of ROS during the aging process^74^. All the molecular systems associated with the increase of oxidative stress during aging are in fact present in these animals^74^ and we report here that cardiac aging in Nfu is associated to a prominent oxidative stress. In the present study, by using human cardiac fibroblasts, we demonstrated for the first time that miR-29 family is regulated by oxidative stress level. Its modulation affected collagen deposition as well as DNA methylation both *in vivo* and *in vitro*. Of note, the knock-down of miR-29 family in zebrafish transgenic animal embryo by expression of a specific sponge (miR-29-sponge)^35^ compromised cardiac function and morphology, enhancing both fibrosis and global DNA methylation. Consistently, transcriptomic analysis revealed a pseudo-hypoxic state in the sponge-29 injected heart. In the heart, hypoxia has been extensively described as detrimental for cardiac performance and homeostasis. A recent study from Watson and co-authors, in fact, demonstrated that hypoxia leads to DNA hypermethylation and fibrosis by collagen deposition^67^. However, other studies demonstrated that hypoxia might play a positive role during cardiac regeneration^75^. This evidence prompted us to investigate miR-29 family expression in cardiac fibroblasts maintained in the presence of 1% O_2_, the standard *in vitro* hypoxic condition. In these experiments, we detected a transient down-modulation of miR-29a and miR-29b leading to collagen deposition and fibrosis, a molecular scenario similar to that observed in the heart of the miR-29-sponge transgenic fish. Noteworthy, transfection of miR-29a or miR-29b mimics or treatment with the DNMT inhibitor RG108 significantly protected cardiac cells from the detrimental effects of hypoxia. In the heart, the relationship among levels of oxidative stress and hypoxia can be crucial to maintain cardiac homeostasis and performance. It has been demonstrated, in fact, that oxidative stress might counteract hypoxia regulating HIF1α degradation by ROS-mediated protein hydroxylation^76^. Hence, we propose here that the physiological accumulation of oxidative stress during aging may control miR-29 family establishing a protective mechanism to limit cardiac fibrosis. Similarly, expression of miR-29 counteracts age-dependent oxidative damage in the brain^35^. Given the up-regulation of miR-29 during aging in multiple organs, this protective action could represent a general phenomenon. Indeed, our results suggest that the miR-29 family strongly affects gene transcription possibly via age-associate DNA methylation changes in Nfu. In consequence, this animal model might represent a system well suitable for investigating the role of miRNAs during aging and specifically that of miR-29 family.

## Methods

### *In vivo* experiments

For expression profiling whole hearts from male animals of Nfu (strain MZM-04/10) and of *D. rerio* (strain Ab) were collected. *N. furzeri* samples were derived from fish at different ages: young (5 weeks), adult (12–21 weeks) and old (27–40 weeks). *D. rerio* hearts were derived from wild type and eGFP-sponge-29 animals, obtained as previously described^35^. To avoid effects of circadian rhythms and feeding, animals were always sacrificed at 10 a.m. in fasted state. For tissue preparation, fish were euthanized with MS-222 and cooled on crushed ice. The protocols of animal maintenance and experiments were approved by the local authority in the State of Thuringia (Veterinaer- und Lebensmittelueberwachungsamt) and by the Italian Ministry of Health (Aut N. 96/2003-A; n°297/2012-A and Aut. N. 1314/2015-PR). Moreover, all researchers who took care of the animals and performed the experiments were appropriately trained by attending specific courses. The whole fish hearts were dissected and transferred into 2 ml tubes with 1 ml cooled QIAzol (Qiagen) and one 5 mm stainless steel bead (Qiagen) was added. Homogenization was performed using a TissueLyzer II (Qiagen) at 20 Hz for 3 × 1 min.

### Cells and treatments

Human cardiac fibroblasts (HCFs) (Innoprot) were grown as previously described^77^. HCFs were exposed 24 h to the following chemicals to induce oxidative stress: 200 µM H_2_O_2_ (Carl Roth) or 10 µM Carbonyl cyanide 3-chlorophenylhydrazone (CCCP; Sigma-Aldrich). 10mM N-Acetyl-L-cysteine (NAC; Sigma-Aldrich) was added to HCF medium 16 h prior H_2_O_2_ or CCCP treatment as ROS scavanger. 50 µM of RG108 (Cayman Chemical) was added to HCF medium as DNMT inhibitor. Differentiated H9C2 rat cardiomyoblasts (RRID: CVCL_0286), derived from the embryonic left ventricle an E13 BDIX female rat heart, were cultured in Dulbecco’s modified Eagle’s medium (DMEM) supplemented with 1% donor calf serum (DCS - Sigma), 1% Pen-Strep (Sigma) and 1% Glutamine (Gibco) to achieve cardiac differentiation.

### Hypoxic protocols

Hypoxic conditions were achieved using a hypoxia chamber at 1% O_2_ (SCI-tive hypoxia workstation, Baker Ruskinn). 48 h later cells were collected for subsequent experiments.

### miRNA overexpression

HCFs were transfected by Lipofectamine RNAiMAX Transfection Reagent (Life Technologies) according manufacture’s instruction with miRVana miRNA mimic for hsa-miR-29a-3p, hsa-miR-29b-3p or scramble (Ambion). After over-night incubation cells were exposed to hypoxia. 48 h later cells were collected for subsequent experiments.

### Quantification of global DNA methylation

DNA was extracted using the E.Z.N.A. DNA tissue kit (VWR OMEGA biotek) according manufacture’s instruction. MethylFlash Methylated (5mC) DNA Quantification Kit was used to quantify methylation status of fish and cell DNA samples according manufacturer’s instruction (Epigentek).

### RNA extraction and qRT-PCR

RNA was extracted from fish and cell samples using Tri-Reagent (SIGMA-ALDRICH) according to manufacturer’s instruction. cDNA synthesis for quantitative real-time PCR (qRT-PCR) was carried out with SuperScript III First-Strand Synthesis Super Mix for qRT-PCR (Invitrogen) according to the manufacturer’s protocol. All reactions were performed in 96-well format in the StepOne Plus Real-Time PCR System (Applied Biosystems) using ORA qPCR Green ROX H Mix (HighQu). For each gene of interest, qRT-PCR was performed as follows: each RNA sample was tested in duplicate and IR, GADPH, or p0 was used to normalize transcript abundance in Nfu, D. Rerio or human cells, respectively. mRNA expression levels were calculated by Comparative Ct Method by using the Applied Biosystem software (Applied Biosystem) and were presented as fold induction of transcripts for target genes. Fold change above 1 denotes upregulated expression, and fold change below 1 denotes downregulated expression versus reference sample. The list of used forward and reverse primers is provided as supplemental Table 6. The sequences were selected based on published sequence data from NCBI database ensemble and NFINgb. All primers were synthesized by Sigma-Aldrich. Primers for miR-16, miR-29a, miR-29b, miR-29c, miR-181c, miR-200a, miR-200b, miR-200c, miR-210, miR-429 and the reagents for reverse transcriptase and qPCR reactions were all obtained from Applied Biosystems. miRNA expression levels in each sample were normalized to miR-16 expression as, under the experimental conditions of the present study, miR-16 was not modulated by aging or treatment in HCF. For Nfu hearts miR-181c was used for normalization of miRNA analysis.

### Sequencing and bioinformatics analysis

For miRNA sequencing total RNA from Nfu heart specimens was extracted as previously described^34^. RNA quality and amount was determined using the Agilent Bioanalyzer 2100 and the RNA 6000 Nano Kit (Agilent Technologies). Library preparation and sequencing was done using Illumina’s NGS platform. One µg of total RNA was used for library preparation using Illumina’s TruSeq small RNA sample preparation kit following the manufacturer’s instruction. The purified libraries were quantified on the Agilent DNA 1000 chip, diluted to 10 nM and subjected to sequencing-by-synthesis on an Illumina HiSeq. 2500 in high-output, 50 bp single-read mode in pools of four or five per lane. Sequencing chemistry v3 was used. Read data were extracted in FastQ format using the Illumina supported tool bcl2fastq v1.8.4. Sequencing resulted in around 8mio 50mio reads per sample with pooling 8 samples per lane. Raw sequencing data were received in FASTQ format. The processing and annotation of small RNA-Seq raw data was performed using the R programming language (version 3.0.2) and the ShortRead Bioconductor package. First, raw data were pre-processed with the following parameters: Quality filtering, eliminating all reads containing an “N”; Adapter trimming, by use of the function trimLRPatterns, allowing up to 2 mismatches and using as adapter sequence “TGGAATTCTCGGGTGCCAAGGAACTCCAGTCAC”. Size filtering removed all the reads with length shorter 18 and longer 33 nucleotides. In a next step reads were aligned, resulting in a direct annotation and quantification. The alignment was divided in two steps, to allow the recognition and the annotation of the reads exceeding reference length. In fact, the algorithm of Bowtie 1.1.2 does not allow aligning longer reads to shorter references. Specifically, first we performed alignment against the reference (*Danio rerio*, miRBase v21) with up to 2 mismatches. In this step the reference used was the mature sequence of microRNAs. Each read was aligned using these criteria with Bowtie 1.0.0 (settings: “-q”, “–threads 8–best”, “—norc”). The remaining reads, which could not align in the previous step, were used as reference for a second alignment step with Bowtie 1.0.0 (settings: -f”, “-a”, “–threads 8–norc”). In this case, the annotated mature microRNAs were aligned against the reads. The information obtained in the two alignment phases was conveyed in one single table, containing a list of all the retrieved sequences and their relative counts. Read counts were normalized to RPM (reads per million). The Bioconductor packages DESeq. 2 was used to identify differentially expressed microRNAs. The non-normalized gene counts have been used here, since the package includes an internal normalization procedure. The resulting p-values were adjusted using the Benjamini and Hochberg’s approach for controlling the false discovery rate (FDR). MicroRNAs with an adjusted p-value < 0.05 were assigned as differentially expressed. For long RNA sequencing, RNA was isolated from 3 zebrafish hearts for each condition (wt and miR-29-sponge) using the miRNeasy micro Kit (Qiagen) combined with on-column DNase digestion (DNase-Free DNase Set, Qiagen) to avoid contamination by genomic DNA. RNA and libraries integrity were verified with a BioAnalyzer 2100 (Agilent) or LabChip Gx Touch 24 (Perkin Elmer). One µg of total RNA was used as input for SMARTer Stranded Total RNA Sample Prep Kit - HI Mammalian (Clontech). Sequencing was performed on the NextSeq. 500 instrument (Illumina) using v2 chemistry, resulting in minimum of 20 M reads per library with 2 × 75 bp paired end setup. The resulting raw reads were assessed for quality, adapter content and duplication rates with FastQC (Available online at http://www.bioinformatics.babraham.ac.uk/projects/fastqc). Trimmomatic version 0.33 was employed to trim reads after a quality drop below a mean of Q20 in a window of 4 nucleotides. Only reads above 20 nucleotides were cleared for further analyses. Trimmed and filtered reads were aligned versus the Ensembl mouse genome version mm10 (GRCm38) using STAR 2.4.2a with the parameter “–outFilterMismatchNoverLmax 0.1” to increase the maximum ratio of mismatches to mapped length to 10%^78^. The number of reads aligning to genes was counted with featureCounts 1.4.5-p1 tool from the Subread package. Only reads mapping at least partially inside exons were admitted and aggregated per gene. Reads overlapping multiple genes or aligning to multiple regions were excluded. Differentially expressed genes were identified using DESeq. 2 version 1.62.25 Only genes with a minimum fold change of ±2, a maximum Benjamini-Hochberg corrected p-value of 0.05, and a minimum combined mean of 5 reads were deemed to be significantly differentially expressed. The Ensemble annotation was enriched with UniProt data (release 06.06.2014) based on Ensembl gene identifiers (Activities at the Universal Protein Resource (UniProt)). The correlation of replicate gene counts was assessed with the Spearman ranked correlation algorithm included in R 3.11 (R: A language and environment for statistical computing). Genes regulated by miR-29 depletion ( ± 0.5 log2 fold change, basemean > 5, fdr < 0.05) derived from mRNASeq of zebrafish samples were imported into the Ingenuity Pathways Analysis Software (Qiagen - Version 39480507) to reveal top disease affected categories by genetic networks. A network is a graphical representation of the molecular relationships between molecules (nodes). The intensity of the node color indicates the degree of up- (orange) or down- (blue) regulation. Nodes are displayed using various shapes that represent the functional class of the gene product. The same list was loaded in the Gene set enrichment analysis software (GSEA) from the Broad Institute. When different genes belonging to a specific set exhibit strong cross-correlation, GSEA boosts the signal-to-noise ratio and permits to detect modest changes in individual genes. Both Sequencing data sets are available on the public repository GEO: Nfu miRNA-Seq at https://www.ncbi.nlm.nih.gov/geo/query/acc.cgi?acc=GSE107062; Zebrafish RNA-Seq at http://www.ncbi.nlm.nih.gov/geo/query/acc.cgi?acc=GSE107003. 

### Western Blot

Western blotting was performed by standard procedures after cell lysis in Laemmli buffer. Nitrocellulose blotting membranes were probed with the following antibodies: HIF1α (Novus biologicals, NB100–479) and α-Tubulin (Cell Signalling, 3873 S). Development was performed by Odyssey CLX reader (LI-COR).

### Histology and Morphometric Analysis

Immunofluorescence and immunohistochemistry were carried out according to standard procedures. Nitrotyrosine (Thermo Fisher) was used according to manufacturer’s instructions, and nuclei were counterstained with DAPI solution. Hematoxylin/eosine-stained sections were prepared to visualize ventricle of wt and miR-29-sponge zebrafish. Fast green-Sirius red staining was performed according manufacturer’s instructions (Chondrex). Immunofluorescence was analysed using a Leica TCS SP8 confocal microscope. Immunohistochemistry was analysed using a Motic AE2000 light microscope (Motic Electric Group Co.) and a plate reader (EnSpire plate reader, PerkinElmer).

### Cardiac Imaging by Echocardiography

In order to assess cardiac function in Wild type and miR-29-sponge zebrafish, animals were anesthetized with low-dose tricaine solution (0.04 mg/mL) and placed in a Petri dish filled with a custom-made sponge, with the ventral side upward. The Petri dish was filled with tricaine medium. Two-dimensional (2D) high-resolution real-time *in vivo* images were obtained with the Vevo2100 Imaging System (VisualSonics), through a 50–70 MHz scanhead. The ventricle was visualized in B-mode modality in a longitudinal plane. The epicardial border was traced in long-axis views from the atrio-ventricular valve annulus to the apex, then back to the annulus, at end-diastole (ED) and end-systole (ES). End-diastolic area (EDA), end-systolic area (ESA) and fractional area change (FAC) were measured; FAC was calculated as follows: (DA − SA)/DA * 100. Echocardiograms were evaluated by two independent examiners blind to the treatment protocol.

### Statistical analysis

Statistical analyses were performed using GraphPad Prism programme. Sample sizes (n) were reported in the corresponding figure legend. No statistical method was used to predetermine sample size. Investigators performing sequencing analysis were blinded during the experiment. All values were presented as mean ± the standard error of the mean (s.e.m.) of at least three independent experiments, unless otherwise indicated. Statistical analyses were performed using non-parametric student’s t-test (unpaired Kolmogorov-Smirnov test) when the comparison has been done between two groups and non-parametric 1-way ANOVA (unpaired Kruskal-Wallis test) for more than 2 groups. For all statistical analysis, a value of p ≤ 0.05 was deemed statistically significant.

All experiments were performed in accordance with relevant guidelines and regulations.

## Electronic supplementary material

Supplemental figures and tables
