# Supplementary material for: Age-dependent increase of oxidative stress regulates microRNA-29 family preserving cardiac health
Source: Sci Rep. 2017 Dec 4;7:16839. doi: 10.1038/s41598-017-16829-w (PMC5715159; doi:10.1038/s41598-017-16829-w)
Supplement: Supplementary file 1 — Supplemental figures and tables [file 41598_2017_16829_MOESM1_ESM.pdf]

Age-dependent oxidative stress increase regulates microRNA-29 family preserving cardiac health

Johanna Heid[1,#], Chiara Cencioni[2,1,#], Roberto Ripa[3,#], Mario Baumgart[4,#], Sandra Atlante[1], Giuseppina Milano[5,6], Alessandro Scopece[5], Carsten Kuenne[7], Stefan Guenther[7], Valerio Azzimato[8], Antonella Farsetti[2], Giacomo Rossi[9], Thomas Braun[7], Giulio Pompilio[5,10], Fabio Martelli[11], Andreas M. Zeiher[12], Alessandro Cellerino[3,*], Carlo Gaetano[1,*], Francesco Spallotta[1,*]

1 Division of Cardiovascular Epigenetics, Department of Cardiology, Goethe University, Frankfurt am Main 60596, Germany; E-Mails: johannaheid@yahoo.de (JH); chcencioni@gmail.com (CC); sandra.atlas@yahoo.it (SA); fspallotta@gmail.com (FS).

2 National Research Council, Institute of Cell Biology and Neurobiology (IBCN), Rome 00143, Italy; E-Mail: antonella.farsetti@cnr.it (AF)

3 Scuola Normale Superiore, Laboratory of Biology (Bio@SNS), c/o Istituto di Biofisica del CNR, Pisa 56124, Italy. E-Mails: roberto.ripa@sns.it (RR); alessandro.cellerino@sns.it (AC)

4 Leibniz Institute on Aging - Fritz Lipmann Institute (FLI), Jena 07745, Germany. E-Mail: Mario.Baumgart@leibniz-fli.de (MB)

5 Vascular Biology and Regenerative Medicine Unit, Centro Cardiologico Monzino-IRCCS, Milan 20128, Italy. E-Mails: gmilano@ccfm.it (GM); ascopece@ccfm.it (AS); Giulio.Pompilio@ccfm.it (GP)

6 Laboratory of Cardiovascular Research, Department of Surgery and Anesthesiology, University Hospital Lausanne, Lausanne 1015, Switzerland.

7 ECCPS Bioinformatics and deep sequencing platform, Max Planck Institute for Heart and Lung Research, Bad Nauheim 61231, Germany; E-Mails: carsten.kuenne@mpi-bn.mpg.de (CK); Stefan.Guenther@mpi-bn.mpg.de (SG); thomas.braun@mpi-bn.mpg.de (TB)

8 Integrated Cardio Metabolic Centre, Department of Medicine, Karolinska Institutet. 141 57 Huddinge, Sweden. E-Mail: valerio.azzimato@gmail.com (VA)

9 Department of Veterinary Sciences, Faculty of Veterinary Medicine, University of Camerino, Camerino (MC) 62032, Italy. E-Mail: giacomo.rossi@unicam.it (GR)

10 Dipartimento di Scienze Cliniche e di Comunità, Università degli Studi di Milano, Milan 20122, Italy

11 IRCCS-Policlinico San Donato, Moleculary Cardiology Laboratory, San Donato Milanese (MI) 20097, Italy. E-Mail: fabio.martelli@grupposandonato.it (FM)

12 Internal Medicine Clinic III, Department of Cardiology, Goethe University, Frankfurt am Main 60596, Germany; E-Mail: zeiher@em.uni-frankfurt.de (AMZ)

Running title: miR-29 protects aging heart

Corresponding author:

Prof. Carlo Gaetano MD

Division of Cardiovascular Epigenetics, Department of Cardiology, Goethe University, Frankfurt am Main 60596, Germany

E-Mail: gaetano@em.uni-frankfurt.de; carlo.gaetano@gmail.com; Tel.: +49-69-6301-87963; Fax: +49-69-6301-86095.

Co-corresponding author:

Francesco Spallotta, PhD

Division of Cardiovascular Epigenetics, Department of Cardiology, Goethe University, Frankfurt am Main 60596, Germany

E-Mail: fspallotta@gmail.com; Tel.: +49-69-6301-87957; Fax: +49-69-6301-86095.

[#] JH, CC, RR and MB co-first authors.

* AC, CG and FS co-senior authors.

Keywords: Aging, oxidative stress, heart, fibrosis and microRNAs

Supplemental figures

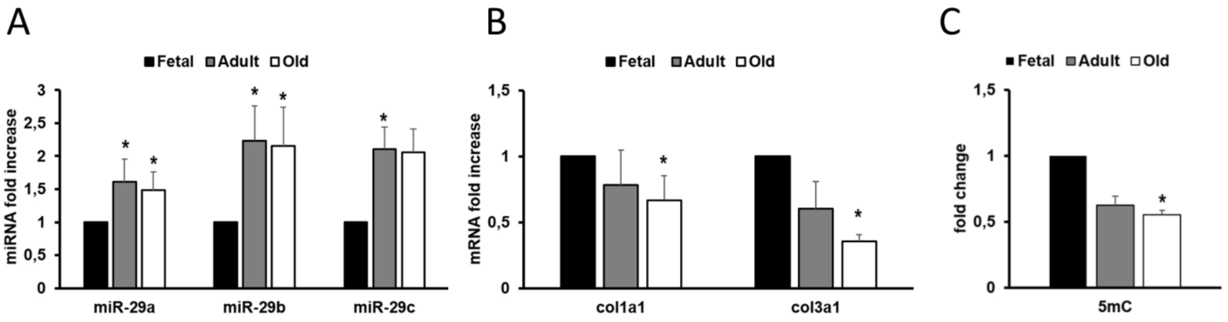

SUPPL. FIG.1. Analysis of miR-29 and its targets in HCF derived from fetal, adult and old donors

(A) qRT-PCR analysis of miR-29 family members in HCFs derived from fetal (black bars), adult (gray bars) and old (white bars) donors expressed as fold-change versus fetal samples (n=3). (B) qRT-PCR analysis of col mRNAs in HCFs derived from fetal (black bars; n=3), adult (gray bars; n=6) and old (white bars; n=6) donors expressed as fold-change versus fetal samples. (C) Global DNA methylation quantification of 5mC in HCFs derived from fetal (black bars; n=3), adult (gray bars; n=3) and old (white bars; n=6) donors. *p<0.05 Vs fetal.

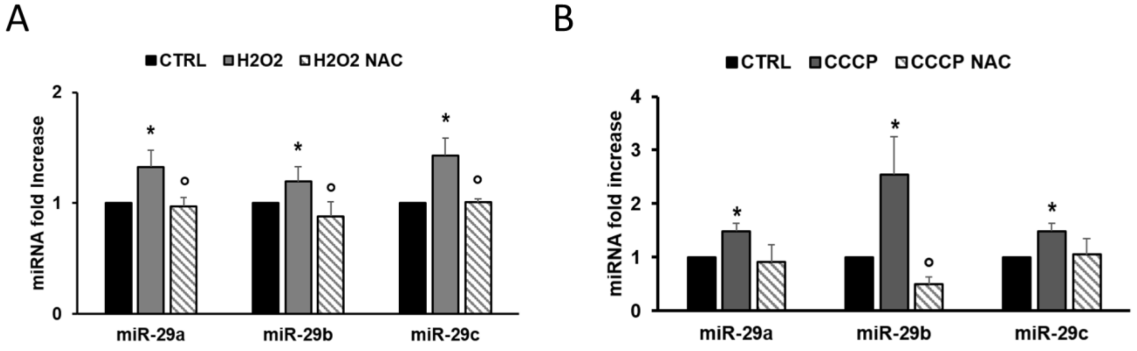

SUPPL. FIG.2 Oxidative stress affects miR-29 family in cardiac cells

(A) qRT-PCR analysis of miR-29 family members in differentiated H9C2 rat cardiomyoblasts cultured in control conditions (CTRL; black bars; n=3), in the presence of H_2O_2 (gray bars; n=3) and of H_2O_2 NAC (striped bars; n=3) expressed as fold-change versus control samples. (B) qRT-PCR analysis of miR-29 family members in HCF prior (CTRL; black bars; n=6) and after treatment with Carbonyl cyanide m-chlorophenyl hydrazine (CCCP; gray bars; n=6) and CCCP NAC (striped bars; n=3) expressed as fold-change versus control samples. *p<0.05 Vs control; °p<0.05 Vs CCCP.

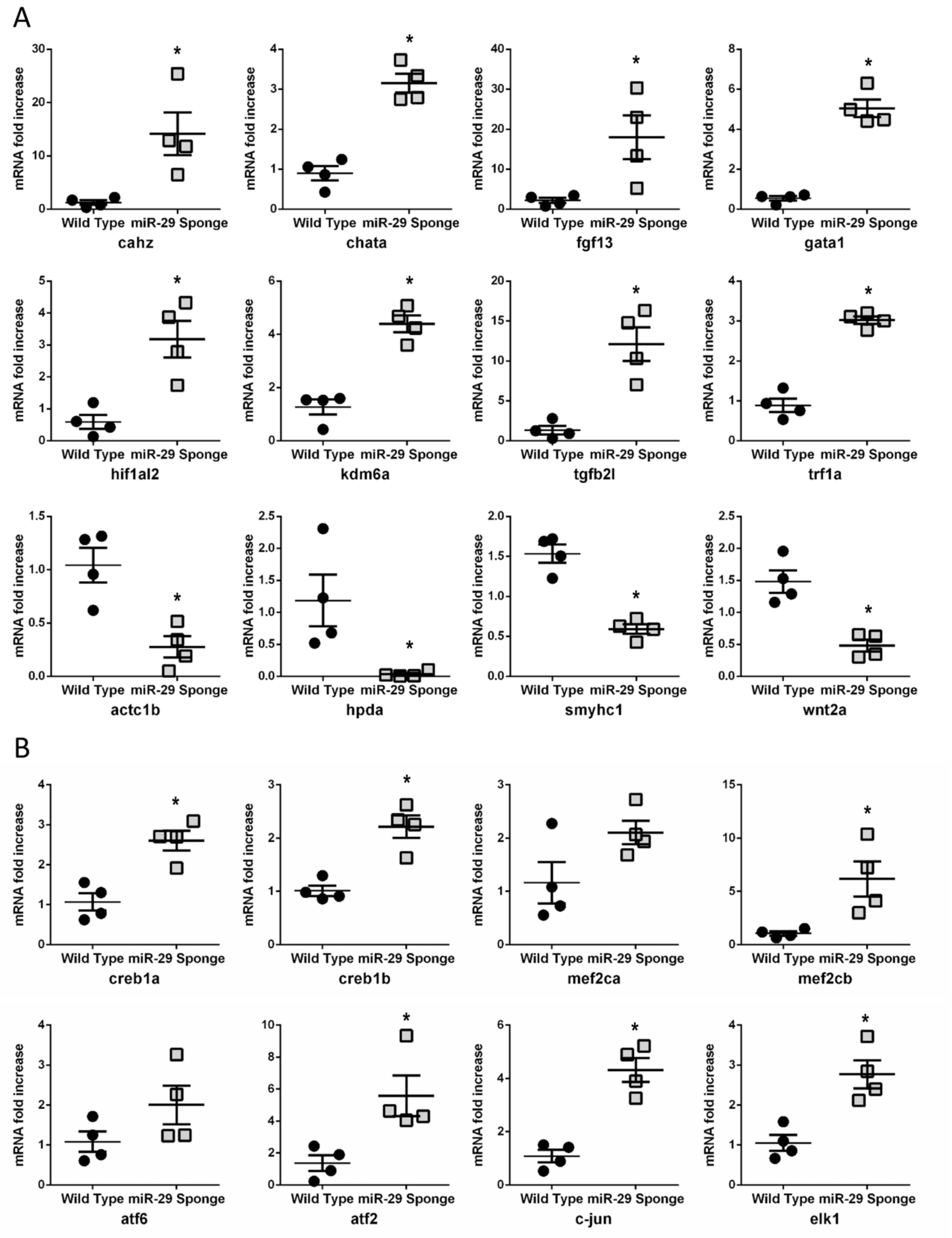
A
mRNA fold increase
Wild Type
miR-29 Sponge
cahz
chata
fgf13
gata1
hif1al2
kdm6a
tgfb2l
trf1a
actc1b
hpda
smyhc1
wnt2a
B
creb1a
creb1b
mef2ca
mef2cb
atf6
atf2
c-jun
elk1

SUPPL. FIG.3 mRNASeq and Ingenuity Pathway analysis validation

(A) qRT-PCR analysis of a set of up and down regulated mRNAs associated with heart, methylation, fibrosis and hypoxia in Wild Type (black circles; n=4) and miR-29-sponge (gray squares; n=4) Zebrafish hearts expressed as fold-change versus Wild Type samples. (B) qRT-PCR analysis of transcription factors upstream of the predicted activation of hypertrophic response revealed by Ingenuity pathway analysis in Wild Type (black circles; n=4) and miR-29-sponge (gray squares; n=4) Zebrafish hearts expressed as fold-change versus Wild Type samples. *p<0.05 Vs Wild Type

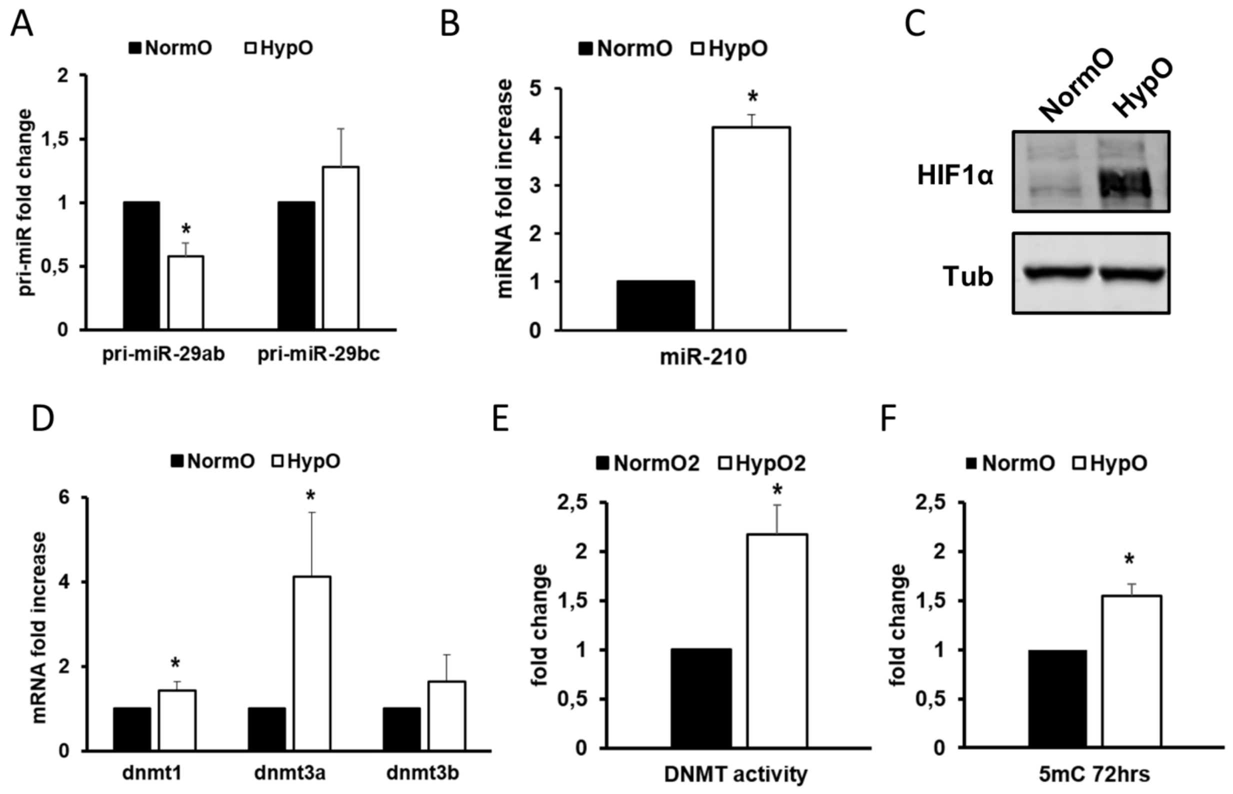

SUPPL. FIG.4 Analysis of hypoxia in HCFs

(A) qRT-PCR analysis of pri-miR-29 in HCFs under normoxic conditions (NormO; black bars) and 48h hypoxia (HypO; white bars), expressed as fold-change versus normoxic samples (n=11). (B) qRT-PCR analysis of miR-210 in HCF under normoxic conditions (NormO; black bars) and 48h hypoxia (HypO; white bars) expressed as fold-change versus normoxic samples (n=3; *p<0.05 Vs normoxia. (C) Representative western blot of hypoxia inducible factor 1α (HIF1α) expression in HCF under normoxic condition and 48h hypoxia. In each condition, α-tubulin was used as loading control. (D) qRT-PCR analysis of dnmts in HCFs under normoxic conditions (NormO; black bars) and 48h hypoxia (HypO; white bars), expressed as fold-change versus normoxic samples (n=11). (E) DNMT activity quantification in HCFs under normoxic condition (black bar) and hypoxia (white bar) expressed as fold-change versus normoxic samples (n=7). (F) Global DNA methylation quantification of 5mC in HCFs cultured in normoxic (black bars) and hypoxic (white bars) conditions (n=3). *p<0.05 Vs normoxia.

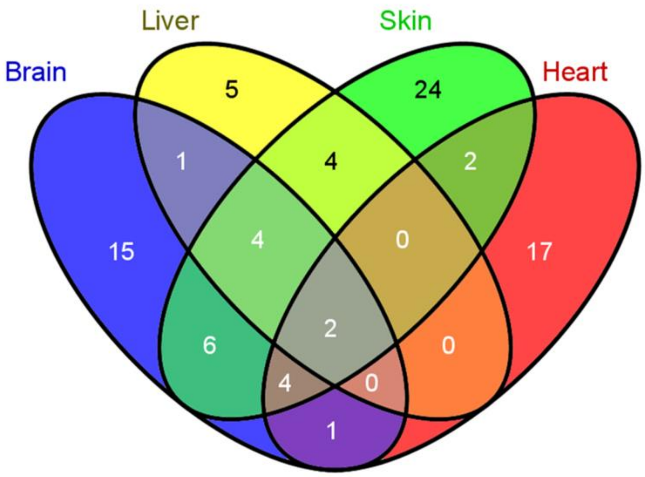

Common in all tissues
miR-29a
miR-27d

SUPPL. FIG.5 miR-29 is up regulated in different Nfu tissues.

Venn diagrams depicting the distribution of up-regulated miRNAs among Nfu heart, brain, skin and liver with age.

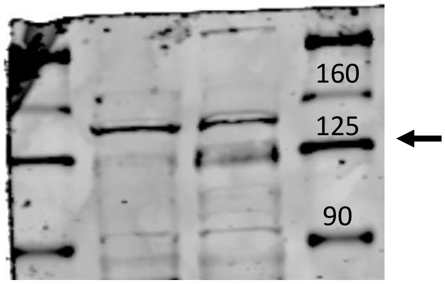

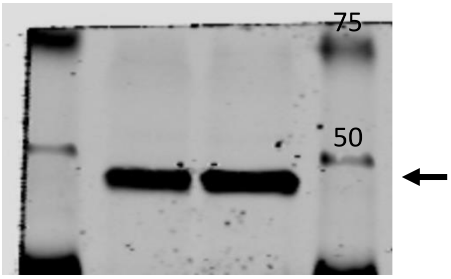

SUPPL. FIG.6 Full-length blot from figure 7 panel E

Representative western blot analysis of hypoxia inducible factor 1α (HIF1α) expression in wild type and miR-29-sponge zebrafish heart. In each condition, α-tubulin was used as loading control.

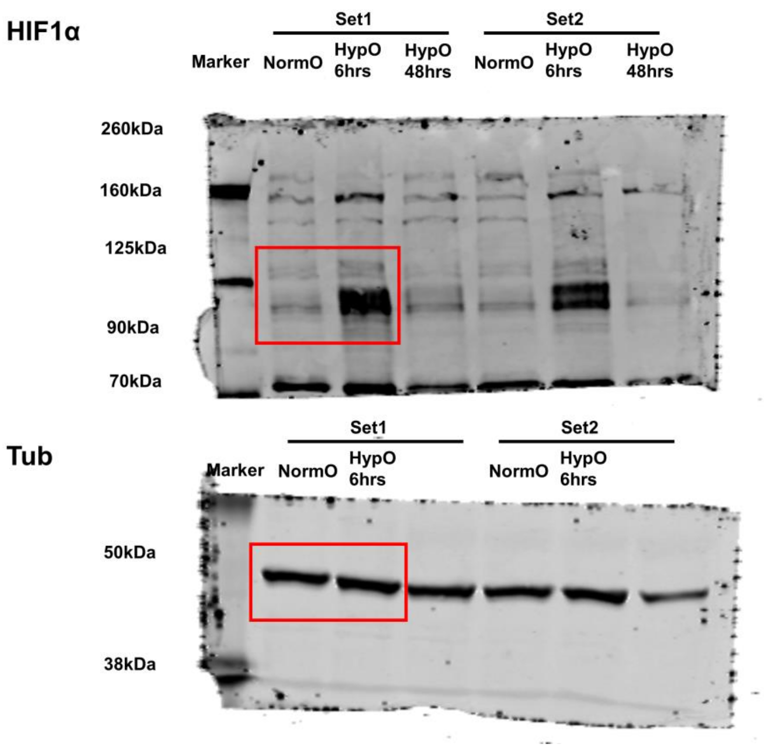

SUPPL. FIG.7 Full-length blot from Suppl. figure 4 panel C

Representative western blot analysis of hypoxia inducible factor 1α (HIF1α) expression in HCF under normoxic condition and 48h hypoxia. In each condition, α-tubulin was used as loading control.

Supplementary table 1. Differentially regulated miRNAs among 5wks, 12 wks and 27wks N. Furzeri depicted in figure 2 panel B.

miR	5wks	12wks	27wks
miR-1	323,1036	636,387264	692,902908
miR-133a-3p	4529,79768	4049,0371	3679,30829
miR-133a-5p	192,249469	180,319	180,243586
miR-499-3p	3008,80428	3568,51655	3481,44867
miR-499-5p	9906,21655	16397,0433	20289,1101
miR-155	61,9113416	20,0572411	150,213746
miR-34a	74,9388183	93,1772622	97,8936591
miR-29a	317,150218	812,008289	948,126539
miR-181b-3p	19,0716094	24,8742793	19,1519694
miR-181b-5p	4610,38585	2401,74467	2534,68659
miRs that are up-regulated (1.3 change fold)			
miR-29b	96,9063022	168,144563	261,564295
miR-133b-3p	146,537847	243,880088	395,561973
miR-2184	918,216772	1877,56678	2468,69029
miR-193a-3p	3,94398564	6,0453357	11,7623056
miR-29a	317,150218	812,008289	948,126539
miR-458-3p	253,11779	640,533336	790,73317
miR-223	79,8547649	179,926058	297,113721
miR-10b-2-3p	0,37970597	0,79936896	1,60644246
miR-730	0,22800113	0,43743851	1,11222556
miR-92b-3p	4,60882739	10,6338358	20,3985311
miR-204-5p	1172,14552	845,747237	2703,05727
miR-429b	0,43719715	1,10873941	3,57090792
miR-458-5p	0	0,38260498	0,8613741
miR-194a	4,9324538	10,3789124	97,0973059
miR-194b	8,01321908	31,1489721	354,234331

miR-459-5p	0,37662542	5,15523475	42,6554603
miRs that are down-regulated (1.3 change fold)			
miR-735-5p	2,61596111	1,46270717	0,14302268
miR-457a	0,38572806	0,37223407	0
miR-203a-3p	274,800144	142,54534	69,7154785
miR-206-3p	42,478648	7,91299281	3,05123364
miR-206-5p	1,62699623	0,14842011	0,07891031
miR-20a-3p	70,6207249	85,828302	67,065456
miR-130a	2,64351471	2,15503416	0,32965821
miR-301c-3p	59,8825229	26,5672123	9,15999434
miR-301c-5p	0,17182915	0,07073388	0
miR-454b	142,106977	109,537074	31,5573926
miR-725-3p	246,33571	137,954791	47,8485642
miR-144-5p	7577,66046	6401,6827	2152,53869
miR-205-5p	1756,43113	1208,38232	395,119796
miR-203a-5p	10,3575767	6,75866996	2,99624985
miR-16a	1687,60014	1126,46326	587,526283
miR-138-3p	1,56733197	1,19259746	0,54545093
miR-203b-5p	27,0078293	18,8271163	8,31013605
miR-181c-5p	238,939	166,194692	95,5201903

Supplementary table 2. List of up- and down-modulated miRNAs in Nfu heart.

(A) Up-regulated miRNAs. **(B)** Down-regulated miRNAs.

(A)

miRNA up-regulated with age
miR-29a
miR-29b
miR-133b-3p
miR-193a-3p
miR-223
miR-10b-2-3p
miR-92b-3p
miR-458-3p
miR-730
miR-2184

(B)

miRNA down-regulated with age
miR-205
miR-181c
miR-725
miR-16
miR-138
miR-203a
miR-203b

Supplementary table 3. Up-regulated transcripts (fdr < 0.05) between WT and miR-29 Sponge zebrafish depicted in figure 7 panel A.

Ensembl gene id	Chr	Start	Stop	Strand	Ensembl gene
ENSDARG00000070494	20	22535507	22576582	-	pdgfra
ENSDARG00000061759	9	33978906	34053238	+	kdm6a
ENSDARG00000052697	5	49114518	49431837	-	fam172a
ENSDARG00000101322	2	5008604	5034301	-	tfr1a
ENSDARG00000017316	13	15631286	15668299	-	bag5
ENSDARG00000071052	23	13159982	13178794	+	si:dkey-150i13,2
ENSDARG00000056725	7	25642032	25647088	+	hmgb3a
ENSDARG00000077236	15	37657985	37806875	+	hspb6
ENSDARG00000098646	5	68943906	68960202	+	mthfd2
ENSDARG00000036629	21	33418568	33422984	+	rps14
ENSDARG00000055290	8	29700878	29704389	-	mpeg1,1
ENSDARG00000090310	22	6892160	6893501	-	CABZ01065328,1
ENSDARG00000037071	23	27748995	27754481	+	rps26
ENSDARG00000042725	8	28414259	28415774	-	cebpb
ENSDARG00000012600	2	42099143	42134146	-	HELZ2
ENSDARG00000079848	18	34205451	34239033	+	gmps
ENSDARG00000091679	3	7344101	7360793	-	CU672268,1
ENSDARG00000004954	3	29783585	29810515	-	grna
ENSDARG00000079839	21	14599906	14666411	-	arrdc1b
ENSDARG00000042533	8	25276021	25282662	+	gstm
ENSDARG00000093182	12	17469912	17481084	-	eif2ak1
ENSDARG00000070839	6	29019766	29095912	-	evi5b
ENSDARG00000030602	16	24274933	24280294	-	rps19
ENSDARG00000103428	10	45301555	45359281	+	ogdhb

ENSDARG00000002405	5	20282967	20310868	-	si:ch211-225b11,1
ENSDARG00000013128	9	3129042	3143876	-	pdk1
ENSDARG00000045636	25	35506531	35539644	-	rbl2
ENSDARG00000038476	2	57777903	57785674	-	si:dkeyp-68b7,7
ENSDARG00000099657	17	1754809	1787403	+	AKT1
ENSDARG00000068710	13	49753659	49826548	-	nid1a
ENSDARG00000011855	8	48976998	49055643	+	aak1a
ENSDARG00000097414	25	28419458	28449680	+	si:ch211-106e7,2
ENSDARG00000006368	22	21127861	21144144	+	crlf1b
ENSDARG00000091260	2	381299	414238	+	mylk4a
ENSDARG00000015053	4	13034769	13256964	-	grip1
ENSDARG00000062148	9	30407082	30437215	-	SENP7
ENSDARG00000053624	21	43846478	43885949	+	csf1rb
ENSDARG00000007708	21	3638418	3645040	-	nsa2
ENSDARG00000068199	18	46265151	46282560	-	pld3
ENSDARG00000040640	22	7729316	7748986	-	si:ch73-44m9,3
ENSDARG00000002956	12	4077636	4108676	+	si:dkey-32n7,4
ENSDARG00000037154	16	34367364	34387992	-	hivep3b
ENSDARG00000043509	19	44185328	44191343	+	rpl11
ENSDARG00000007693	17	12788568	12792670	+	nfkbiab
ENSDARG00000095783	6	49762576	49770447	-	si:dkey-249d8,1
ENSDARG00000013110	10	20150231	20246676	+	dmtn
ENSDARG00000033170	12	624354	640031	-	sult2st1
ENSDARG00000028228	13	11303655	11312559	+	zbtb18
ENSDARG00000015536	7	26980282	27181168	+	sox6
ENSDARG00000017034	25	32062320	32084406	+	sqrdl
ENSDARG00000098257	13	41044796	41420889	-	pcdh15a
ENSDARG00000095825	23	46042411	46053109	-	si:ch73-67c22,3
ENSDARG00000006003	13	35778503	35782443	-	mycla

ENSDARG00000104760	16	35641749	35692535	+	MAP3K6
ENSDARG00000092310	19	43984189	44008335	+	si:ch211-199g17,2
ENSDARG00000006900	8	26040453	26060002	+	impdh2
ENSDARG00000062134	23	21140838	21188181	+	kcnab2b
ENSDARG00000042630	20	30991214	30998155	-	hebp2
ENSDARG00000025766	23	27114570	27134531	-	DTX3
ENSDARG00000101362	22	4733941	4738182	+	mibp
ENSDARG00000042018	9	6600595	6624003	+	fhl2a
ENSDARG00000077499	16	38065318	38117920	-	plekho1b
ENSDARG00000025850	23	9285466	9293608	+	rps21
ENSDARG00000037421	14	21035277	21039140	-	egr1
ENSDARG00000086762	19	20453534	20470242	+	dfna5a
ENSDARG00000031954	22	10115371	10135764	-	rbck1
ENSDARG00000016156	12	46867600	46921771	+	fam53b
ENSDARG00000059857	2	50110400	50126493	-	blvra
ENSDARG00000071697	14	20621456	20703366	+	zgc:66433
ENSDARG00000075977	25	20977374	20994568	-	prr5
ENSDARG00000036840	11	11532157	11534848	-	krt15
ENSDARG00000022165	4	12720116	12726664	-	mgst1,2
ENSDARG00000101361	2	22850713	22875318	-	znf644a
ENSDARG00000104311	16	35603026	35634437	-	scmh1
ENSDARG00000071413	22	21491753	21520262	+	mier2
ENSDARG00000061140	2	31399372	31451940	+	colec12
ENSDARG00000005540	10	20112739	20171071	+	xpo7
ENSDARG00000060120	6	43022205	43031360	-	glyctk
ENSDARG00000006526	1	4806323	4845260	-	fn1b
ENSDARG00000040930	16	14697920	14785787	+	deptor
ENSDARG00000003991	6	14948853	14974537	-	fhl2b
ENSDARG00000020979	6	51676228	51763504	+	fam65c

ENSDARG00000058731	8	12816821	12829697	-	slc2a6
ENSDARG00000057949	14	16819951	16856628	+	slc43a3b
ENSDARG00000105308	16	34446776	34471531	-	si:ch211-12b16,1
ENSDARG00000078676	25	3772248	3853924	-	myrf
ENSDARG00000036893	7	8024805	8098285	-	f13a1b
ENSDARG00000019280	21	41005607	41047237	-	larsb
ENSDARG00000100293	5	53464326	53514388	-	arhgef15
ENSDARG00000075054	10	3081322	3142059	-	RASGRF2
ENSDARG00000094451	8	23651015	23681186	-	cfp
ENSDARG00000003655	22	21103135	21125867	-	tmem59l
ENSDARG00000069388	7	21521169	21540116	+	tmem88b
ENSDARG00000029473	8	25279930	25282663	+	zgc:173994
ENSDARG00000020811	21	25551831	25568004	-	efemp2b
ENSDARG00000060196	12	46934399	46943805	-	lhpp
ENSDARG00000020761	11	13466845	13476123	+	arrdc2
ENSDARG00000044183	10	17979007	17988729	+	prkab1a
ENSDARG00000044191	6	44166191	44196204	+	gxylt2
ENSDARG00000030340	2	3186685	3203882	-	guk1a
ENSDARG00000102202	6	3550045	3552274	+	phospho2
ENSDARG00000098295	16	23172294	23274851	+	efna3b
ENSDARG00000074782	7	64707696	64729489	+	ADGRG5
ENSDARG00000041982	10	11635566	11806389	-	adamts6
ENSDARG00000030630	13	35766544	35828850	+	mfsd2aa
ENSDARG00000011376	22	5652789	5670636	+	zgc:77816
ENSDARG00000016676	18	22804399	22987598	+	gnao1a
ENSDARG00000091650	15	37534845	37552568	+	igflr1
ENSDARG00000059001	8	8889285	8895484	+	pim2
ENSDARG00000071361	22	17430145	17434609	-	si:ch211-197g15,10
ENSDARG00000036966	3	32612232	32646536	+	hsd3b7

ENSDARG00000053800	15	41736239	41751261	-	si:ch211-276c2,4
ENSDARG00000103370	2	22839623	22846600	+	lrrc8da
ENSDARG00000102693	7	21519257	21572102	-	si:dkey-85k7,8
ENSDARG00000068759	5	13977065	14000405	-	CDCA2
ENSDARG00000010008	24	32261512	32278038	+	vim
ENSDARG00000059646	8	23617885	23635491	+	nt5dc2
ENSDARG00000071879	14	8464038	8488967	-	tgfb2l
ENSDARG00000079933	10	26540621	26551029	-	SLC46A3
ENSDARG00000092281	23	19679948	19726572	+	FLNB
ENSDARG00000068820	5	13147071	13152470	+	h2afva
ENSDARG00000090830	5	59845417	60013852	+	tmem132e
ENSDARG00000096508	20	12205588	12209943	+	si:ch211-265g21,1
ENSDARG00000095298	15	41789324	41791968	-	ftr73
ENSDARG00000095962	23	45189342	45204744	-	fhdc5
ENSDARG00000076858	10	20299073	20299794	+	C8orf4
ENSDARG00000092945	19	37922518	37932627	+	si:ch211-250g4,3
ENSDARG00000021539	11	27970967	28177012	+	ephb2b
ENSDARG00000096331	3	32468105	32471477	-	si:ch73-248e21,7
ENSDARG00000023058	17	6635771	6653555	+	foxo3a
ENSDARG00000009336	5	33949212	33975349	+	aif1l
ENSDARG00000071013	6	13114313	13142615	-	arl6ip6
ENSDARG00000027491	12	48750693	48759117	-	uros
ENSDARG00000092067	16	31890786	31913397	+	atn1
ENSDARG00000015149	8	25610583	25624793	+	wasa
ENSDARG00000079564	2	24600869	24614260	-	vmhc
ENSDARG00000059049	19	5772681	5781451	-	CLEC4A
ENSDARG00000088116	8	25283033	25287875	+	gstm3
ENSDARG00000014179	23	27627245	27645126	-	pfkma
ENSDARG00000038587	2	45306857	45309466	+	CU929150,1

ENSDARG00000086947	25	13087219	13092173	-	si:ch211-147m6,1
ENSDARG00000044167	6	47845001	47867099	+	padi2
ENSDARG00000086724	6	47485066	47507826	-	im:7151449
ENSDARG00000059093	5	68156722	68270435	-	ANK1
ENSDARG00000077326	16	30950355	30974606	-	slc45a4
ENSDARG00000035415	5	34798592	34801415	+	ptger4b
ENSDARG00000044212	19	37955768	37979726	+	CR735126,1
ENSDARG00000090396	7	5183672	5195280	-	CABZ01074946,1
ENSDARG00000088444	20	4078267	4089231	+	sell
ENSDARG00000062687	6	10214377	10341428	+	KCNH7
ENSDARG00000040536	15	41754331	41756933	-	si:ch211-276c2,5
ENSDARG00000023713	2	2895633	2907365	+	aqp1a,1
ENSDARG00000088967	3	22312765	22416759	+	WNK4
ENSDARG00000105154	KN150699,1	15689	19506	+	creg1
ENSDARG00000058839	5	8741820	8773690	+	SUSD1
ENSDARG00000042835	15	4430650	4537240	-	tfdp2
ENSDARG00000020114	8	31047865	31065783	-	slc20a1a
ENSDARG00000037373	23	21876859	22020056	-	ephb2a
ENSDARG00000086775	23	2810969	2842325	+	top1
ENSDARG00000091368	16	34346314	34352311	-	si:dkey-18h14,2
ENSDARG00000073743	8	50202096	50229269	+	slc25a37
ENSDARG00000104047	11	14222939	14234303	+	si:ch211-262i1,3
ENSDARG00000042090	5	1646191	1681536	+	PLA2G4C
ENSDARG00000100677	3	14349275	14358961	+	tmem205
ENSDARG00000076657	23	7653868	7740872	-	plagl2
ENSDARG00000055991	15	23576105	23594680	-	hmbsb
ENSDARG00000001889	23	35609927	35612595	+	tuba1a
ENSDARG00000095167	10	20186416	20193947	-	si:dkeyp-66d7,4
ENSDARG00000062854	1	10436884	10833134	-	sdk1b

ENSDARG00000095509	5	20039192	20042151	+	si:dkeyp-91h9,1
ENSDARG00000096539	20	22294520	22297494	+	si:ch211-278f21,4
ENSDARG00000014866	25	36502300	36513143	-	hprt1l
ENSDARG00000007349	5	44344664	44391191	+	dmrt1
ENSDARG00000013539	13	15826890	15863480	-	ikzf1
ENSDARG00000093851	8	12413746	12631408	-	si:ch73-332k15,1
ENSDARG00000092652	21	16048284	16070514	+	qrfpr4
ENSDARG00000020771	2	35258307	35491658	+	tnr
ENSDARG00000029019	16	26576173	26614727	+	epb41b
ENSDARG00000003027	16	29750934	29755589	-	scnm1
ENSDARG00000018146	11	35786596	35894988	-	gpx1a
ENSDARG00000069282	15	23809952	23826315	-	bbc3
ENSDARG00000019930	22	16509305	16519060	+	tal1
ENSDARG00000010425	20	1333869	1363090	-	scara5
ENSDARG00000075557	6	49652223	49674823	-	apcdd1l
ENSDARG00000039373	9	16437405	16619849	+	epha3
ENSDARG00000019525	5	62554860	62592686	-	tbcelb
ENSDARG00000086579	1	50998008	51016982	+	APLF
ENSDARG00000041257	14	16802350	16816060	-	smtnl1
ENSDARG00000061747	9	34054338	34068291	-	C9HXorf36
ENSDARG00000034588	3	22142529	22186270	+	scn4ab
ENSDARG00000004392	5	26804793	26814928	+	hdr
ENSDARG00000044326	6	36486571	36492212	+	BX950188,1
ENSDARG00000034852	10	17502924	17561599	-	nt5c2l1
ENSDARG00000095412	8	25320378	25322349	+	si:dkey-183p4,9
ENSDARG00000098508	KN150067,1	4416	13245	-	SEMA7A
ENSDARG00000045999	7	12688763	12703714	+	saa
ENSDARG00000095019	18	38307869	38315657	+	lmo2
ENSDARG00000053646	8	37667755	37706317	+	adrb3a

ENSDARG00000101146	11	44774774	44784684	-	CR293511,2
ENSDARG00000078781	6	53373119	53391348	-	si:ch211-161c3,6
ENSDARG00000074441	8	4519426	4529990	-	gp1bb
ENSDARG00000095304	5	41671657	41672397	-	si:ch211-207c6,2
ENSDARG00000040485	16	21619295	21635407	+	dfna5b
ENSDARG00000076950	16	17352455	17392053	-	kel
ENSDARG00000096917	22	13251916	13325579	-	si:ch211-227m13,1
ENSDARG00000101879	6	39536017	39540467	+	CU467856,1
ENSDARG00000091723	17	45619388	45638271	+	si:ch211-202f3,4
ENSDARG00000043126	22	9887325	9894010	+	blf
ENSDARG00000097160	9	7262505	7279486	-	si:ch211-121j5,4
ENSDARG00000042925	22	15225144	15254255	-	mfng
ENSDARG00000070214	11	37681634	37730214	-	slc41a1
ENSDARG00000086708	8	25323757	25332556	+	TRAF3IP3
ENSDARG00000090834	3	14394007	14414315	+	epor
ENSDARG00000074231	3	13114701	13197182	+	SLC9A3R2
ENSDARG00000062707	8	2202369	2247934	-	plat
ENSDARG00000070894	23	19728958	19741415	+	abhd6b
ENSDARG00000074581	5	13281751	13335244	-	add2
ENSDARG00000005704	8	25288864	25310828	-	eps8l3
ENSDARG00000013477	11	25174298	25181240	-	gata1a
ENSDARG00000077121	7	25052585	25077440	+	cyp26b1
ENSDARG00000078839	10	18505004	18506503	-	LRRTM4
ENSDARG00000017429	18	46397891	46466483	-	sphkap
ENSDARG00000063370	23	4385724	4409136	+	sgk2a
ENSDARG00000058818	23	6652495	6698134	+	rbm38
ENSDARG00000096829	18	46153487	46162090	+	blvrb
ENSDARG00000030262	23	9973771	9990598	-	si:ch211-220i18,4
ENSDARG00000018461	24	7712202	7717159	+	FTL

ENSDARG00000059682	1	43440003	43460114	-	slc43a3a
ENSDARG00000079947	21	17243516	17259903	-	gfi1b
ENSDARG00000075830	3	30384523	30494377	-	syt3
ENSDARG00000012137	20	5063655	5094363	+	cyp46a1,1
ENSDARG00000103555	3	12696674	12698948	+	si:ch211-8c17,4
ENSDARG00000061848	2	24281303	24357609	-	col15a1b
ENSDARG00000088505	23	12217089	12223715	-	fam217b
ENSDARG00000060862	16	25640277	25653480	-	atxn1b
ENSDARG00000054447	11	43142747	43165191	+	SLC29A1
ENSDARG00000044550	21	27290747	27303205	-	hif1al2
ENSDARG00000062530	19	19825871	19834636	+	ASTE1
ENSDARG00000073781	21	25508277	25519545	-	si:dkey-17e16,9
ENSDARG00000090638	23	4753753	4756677	+	gp9
ENSDARG00000056765	8	21334686	21340400	-	ela2l
ENSDARG00000011672	8	26564692	26623305	-	sema3b
ENSDARG00000002194	13	45357468	45387357	-	rhd
ENSDARG00000091101	17	53310101	53311981	+	fabp10b
ENSDARG00000097339	17	53309479	53309937	+	slc9a1
ENSDARG00000004647	24	21185626	21199022	-	spice1
ENSDARG00000103576	22	35985725	36013476	+	EPHB3
ENSDARG00000020984	20	133488	158943	-	slc16a10
ENSDARG00000101814	22	24617696	24639563	+	lpar3
ENSDARG00000099600	11	44760126	44771398	-	si:dkey-93h22,7
ENSDARG00000010764	15	43285404	43315792	+	flj13639
ENSDARG00000036558	9	42019826	42177718	+	col18a1
ENSDARG00000053026	12	38541303	38630908	+	kif19
ENSDARG00000009626	24	33311911	33361719	-	nrbp2b
ENSDARG00000096355	3	33833739	33965713	-	ighv1-4
ENSDARG00000055642	12	14040794	14092413	+	GPT

ENSDARG00000079252	12	9811633	9838233	+	fam117ab
ENSDARG00000036587	1	46474610	46482000	+	cbr1
ENSDARG00000097679	15	41780517	41787133	-	si:dkey-101o7,6
ENSDARG00000095587	5	41652056	41653160	-	si:ch211-207c6,15
ENSDARG00000042041	5	8843646	8845563	+	tal2
ENSDARG00000102157	4	1089710	1134207	-	si:ch211-117i20,2
ENSDARG00000069031	21	40993682	41002293	-	plac8l1
ENSDARG00000098612	4	1132898	1271224	+	chrm2a
ENSDARG00000022518	6	29486372	29621708	-	pex5la
ENSDARG00000071876	14	9150661	9169831	+	rell2
ENSDARG00000031119	3	61168723	61258261	-	baiap2l1b
ENSDARG00000054914	16	30990524	31042800	-	dgat1b
ENSDARG00000094215	4	75411477	75428073	-	si:dkey-240n22,2
ENSDARG00000060569	17	37677119	37702243	+	tmem229b
ENSDARG00000057206	12	15624642	15633978	+	nmt1b
ENSDARG00000011257	16	9718819	9771303	+	enpp2
ENSDARG00000061918	14	25242349	25279919	-	cplx2
ENSDARG00000009131	20	23532385	23541855	-	slc10a4
ENSDARG00000098045	5	49103746	49122419	+	si:dkey-172f14,2
ENSDARG00000096849	3	32193429	32205981	-	si:dkey-16p21,8
ENSDARG00000076142	19	33616996	33624863	-	trib1
ENSDARG00000086703	23	31993314	32005545	-	si:dkey-126g1,7
ENSDARG00000076093	15	41758263	41798441	-	ftr76
ENSDARG00000058725	21	11737208	11741824	-	rfesd
ENSDARG00000045144	12	20214196	20215400	+	HBZ
ENSDARG00000087706	16	22004166	22006416	-	dicp3,3
ENSDARG00000090757	7	8124164	8124803	+	CR382283,1
ENSDARG00000056797	1	13592828	13620325	+	ppp2r2ca
ENSDARG00000077736	14	50866586	50892594	-	CPLX1

ENSDARG00000004158	17	44666401	44809896	+	tmem63c
ENSDARG00000077881	23	11412343	11513888	+	chl1a
ENSDARG00000045316	24	23975598	24018027	-	map7d2b
ENSDARG00000100898	4	1178079	1178196	+	5S_rRNA
ENSDARG00000077367	8	11953547	12074952	+	ntng2a
ENSDARG00000063475	15	5037461	5083836	+	abcg1
ENSDARG00000055373	11	35186568	35313808	-	sema3fb
ENSDARG00000038643	8	21321758	21338179	+	alas2
ENSDARG00000023952	20	18840935	18857670	+	fam167ab
ENSDARG00000087745	11	28229124	28267804	+	LRRC38
ENSDARG00000099351	20	6818827	6822741	-	igfbp1a
ENSDARG00000103978	16	36118244	36154631	+	sh3bp5b
ENSDARG00000068096	5	29814097	29818483	-	atf5a
ENSDARG00000019253	20	14118833	14158103	-	rhag
ENSDARG00000017400	6	7894004	7905419	+	klf1
ENSDARG00000070579	1	2299202	2305391	-	ggact,3
ENSDARG00000007220	15	18431302	18638742	-	ncam1b
ENSDARG00000068082	6	60025410	60027562	-	wbp4
ENSDARG00000028850	15	18262856	18273701	-	btr16
ENSDARG00000069593	9	30399649	30402925	+	senp7a
ENSDARG00000078429	22	24703538	24830407	-	vtg4
ENSDARG00000097651	6	60039386	60046179	+	tgm8
ENSDARG00000096667	1	25974158	25976405	+	si:dkey-25o16,2
ENSDARG00000020890	16	29716075	29749570	+	tmod4
ENSDARG00000077840	20	10111869	10133202	-	meis2b
ENSDARG00000104027	3	44061195	44070142	+	si:ch211-207b24,4
ENSDARG00000098113	15	29962216	29984669	-	WSCD1
ENSDARG00000011166	2	29493850	29501875	-	cahz
ENSDARG00000052376	1	52836283	52844567	-	b3gnt2a

ENSDARG00000043089	15	1002489	1015550	+	alox5b,2
ENSDARG00000033355	5	1612139	1636508	+	PLA2G4C
ENSDARG00000070857	23	20142356	20174093	-	si:dkey-32e6,6
ENSDARG00000035056	14	31843664	32063455	-	fgf13a
ENSDARG00000015072	5	44407809	44410963	+	dmrt2a
ENSDARG00000077504	8	29709460	29710161	-	si:ch211-103n10,5
ENSDARG00000053066	6	49883211	49889765	+	tubb1
ENSDARG00000052016	1	43425989	43440139	+	cabp2a
ENSDARG00000040741	2	44552954	44657754	+	kcnab1b
ENSDARG00000084957	21	27298430	27298566	+	5S_rRNA
ENSDARG00000063436	10	3481035	3514892	+	RPH3A
ENSDARG00000012881	3	19916121	19944785	-	slc4a1a
ENSDARG00000075713	15	2218531	2224850	-	shox2
ENSDARG00000052375	1	52817984	52833422	-	zgc:66455
ENSDARG00000037478	7	42794171	42972949	-	cdh8
ENSDARG00000070317	16	25907423	25914747	-	irge4
ENSDARG00000093971	23	11409649	11410803	-	si:dkey-177g18,1
ENSDARG00000016825	22	24783772	24790442	-	vtg6
ENSDARG00000103883	22	1106212	1110896	+	si:ch1073-181h11,2
ENSDARG00000007739	1	1564308	1667865	+	atp1a1a,2
ENSDARG00000079637	21	10773793	10787937	+	alpk2
ENSDARG00000088641	19	40609342	40611978	-	grn2
ENSDARG00000092126	22	24723477	24830407	-	vtg5
ENSDARG00000055809	22	24842066	24853200	-	vtg2
ENSDARG00000059747	3	38651641	38717447	+	si:dkey-106c17,3
ENSDARG00000061585	14	29635046	29751221	+	cyp4v7
ENSDARG00000097522	6	18067656	18068466	+	si:ch211-89i1,3
ENSDARG00000105160	13	18106014	18109699	+	BX927098,1
ENSDARG00000028548	17	7367211	7374391	+	klhl10b,2

ENSDARG00000092949	2	15121704	15129715	-	si:ch73-366i20,1
ENSDARG00000061014	18	48613669	48645587	+	kcnj5
ENSDARG00000038248	1	2302346	2305380	-	ggact,2
ENSDARG00000092233	22	24703341	24710564	-	vtg1
ENSDARG00000092419	22	24703538	24830426	-	vtg7
ENSDARG00000015854	13	29278909	29290881	-	chata

Supplementary table 4. Down-regulated transcripts (fdr < 0.05) between WT and miR-29 Sponge zebrafish depicted in figure 7 panel A.

Ensembl gene id	Chr	Start	Stop	Strand	Ensembl gene
ENSDARG00000090870	21	43635262	43641455	-	si:ch1073-263o8,2
ENSDARG00000093487	3	4037556	4045078	-	si:dkey-217f16,2
ENSDARG00000080001	22	27489648	27499674	-	si:dkey-208m12,2
ENSDARG00000099482	13	15719189	15723933	+	BX569782,1
ENSDARG00000014480	21	8106127	8169341	+	nr6a1b
ENSDARG00000092924	24	9871275	9874126	-	zgc:171474
ENSDARG00000037473	16	26259509	26267326	-	cd79a
ENSDARG00000099364	22	7492685	7505123	-	BX511034,4
ENSDARG00000095281	24	9841893	9850767	-	zgc:152652
ENSDARG00000078256	21	25588421	25594777	+	OVOL1
ENSDARG00000056443	3	2452805	2459471	-	zgc:152753
ENSDARG00000039243	13	37491630	37494400	-	zgc:152791
ENSDARG00000099197	17	117124	124703	-	actc1b
ENSDARG00000089602	3	4032082	4043655	-	si:dkey-217f16,1
ENSDARG00000099243	15	32885186	32889721	+	CR855389,1
ENSDARG00000100333	7	71156956	71164331	-	FO818712,1
ENSDARG00000053254	3	32681334	32686893	-	mylpfa
ENSDARG00000059139	12	798373	802502	+	WFIKKN2
ENSDARG00000045423	24	9887515	9890368	+	BX571823,1
ENSDARG00000088141	3	58785150	58790211	-	CU570684,4
ENSDARG00000074749	9	40883826	41043134	-	abca12
ENSDARG00000086048	3	3418659	3427179	-	si:ch211-229i14,2
ENSDARG00000094171	22	14336629	14451352	-	lrp1ba
ENSDARG00000040534	13	8508749	8511340	+	epcam
ENSDARG00000012311	7	69588478	69616337	-	myoz2a

ENSDARG00000097080	12	38809488	38856958	+	si:ch73-181m17,1
ENSDARG00000054103	21	37919751	37926113	-	ripply1
ENSDARG00000041433	20	46135871	46139685	+	si:dkey-7c18,24
ENSDARG00000067567	25	1710156	1736007	-	SLC6A13
ENSDARG00000069251	20	43839944	43846622	-	si:dkeyp-50f7,2
ENSDARG00000090015	3	2194988	2237911	+	BX321875,1
ENSDARG00000091715	7	781251	815299	+	si:dkey-162h11,2
ENSDARG00000083753	6	54074414	54074548	+	5S_rRNA
ENSDARG00000063347	12	4361435	4397134	-	TRPM4
ENSDARG00000036237	18	62932	73844	+	slc27a2a
ENSDARG00000088937	10	35397206	35419988	+	ADGRG2
ENSDARG00000103392	11	12187674	12187970	+	Metazoa_SRP
ENSDARG00000038428	24	19374132	19443129	+	sulf1
ENSDARG00000097727	15	22262127	22267507	-	si:ch211-286b4,3
ENSDARG00000034757	14	30086638	30111904	-	zdhhc2
ENSDARG00000016112	14	1342084	1349806	+	bbs12
ENSDARG00000101380	5	56971337	56980781	+	cryabb
ENSDARG00000008849	18	6179082	6266244	+	ptprq
ENSDARG00000101901	5	56951195	56953707	-	zgc:193711
ENSDARG00000102713	4	69248343	69268517	-	si:ch211-205a14,1
ENSDARG00000077673	15	41962105	41973354	+	wu:fb15h11
ENSDARG00000077134	24	6365857	6472080	-	gpr158a
ENSDARG00000010957	12	10161281	10262258	-	mpp2b
ENSDARG00000018351	5	2544820	2582076	+	hpda
ENSDARG00000103324	4	68765919	69053224	+	si:dkey-254e13,6
ENSDARG00000096775	20	24232973	24239462	+	si:ch211-225h9,2
ENSDARG00000097611	22	34652091	34678717	+	si:ch73-304f21,2
ENSDARG00000098881	11	30904132	30915517	-	si:dkey-238i5,3
ENSDARG00000078976	14	4810207	4811015	+	nanos1

ENSDARG00000013022	22	7664593	7670333	-	si:ch211-59h6,1
ENSDARG00000093349	9	19870700	20052181	-	si:ch211-141e20,2
ENSDARG00000052450	5	56958990	56971040	-	hspb2
ENSDARG00000074052	1	48880007	48896858	-	si:ch211-281g13,5
ENSDARG00000063435	12	4358014	4497181	-	trpm4b,2
ENSDARG00000075864	10	19069384	19135185	+	igsf9a
ENSDARG00000087133	11	9823657	10001798	-	nlgn1
ENSDARG00000096904	23	16556526	16558847	-	si:dkeyp-100a5,4
ENSDARG00000093214	5	37593300	37596927	-	si:ch211-284e13,9
ENSDARG00000054096	21	37965371	37983432	-	rbm41
ENSDARG00000076826	24	6658375	6868565	-	dpp6a
ENSDARG00000021455	10	9878451	10059882	-	strbp
ENSDARG00000090468	4	6563794	6572040	+	ppp1r3aa
ENSDARG00000086628	3	56534393	56549661	-	stk19
ENSDARG00000027618	22	31258604	31517396	+	grip2b
ENSDARG00000103026	22	24362719	24455169	+	p3h2
ENSDARG00000069467	21	24658367	24828849	-	igsf9bb
ENSDARG00000071001	19	7919719	7942355	+	NUGGC
ENSDARG00000102123	4	223161	240969	-	phtf2
ENSDARG00000087013	24	32120983	32235633	-	cubn
ENSDARG00000098176	21	2077340	2124410	-	si:rp71-1h20,5
ENSDARG00000103590	23	42350569	42364992	+	cyp2aa6
ENSDARG00000023822	7	14946420	15004253	-	zgc:73349
ENSDARG00000088959	9	19812494	19846653	+	pdxka
ENSDARG00000098204	9	561485	582765	+	CU984600,2
ENSDARG00000078404	23	970881	1008485	-	CDH26
ENSDARG00000052958	8	48620752	48621775	+	nppb
ENSDARG00000056453	21	30381339	30431210	-	afap1l1b
ENSDARG00000001470	1	46334486	46430897	-	mhc1zea

ENSDARG00000052097	3	5628956	5645579	-	trim35-31
ENSDARG00000021688	1	6878883	6898529	-	mxa
ENSDARG00000061685	18	1112470	1251622	-	hcn4
ENSDARG00000099959	24	40956288	40968424	-	smyhc1
ENSDARG00000094984	22	17442088	17472080	-	si:ch211-197g15,8
ENSDARG00000102019	21	2112832	2116485	-	si:rp71-1h20,3
ENSDARG00000041092	18	20880857	20886357	+	pgpep1l
ENSDARG00000105088	15	10079804	10533156	-	tenm4
ENSDARG00000102356	23	41935799	41959885	+	scp2b
ENSDARG00000005050	6	48254690	48272928	+	wnt2ba
ENSDARG00000087844	23	23305585	23326117	+	plekhn1
ENSDARG00000077468	24	5176384	5183153	+	si:ch73-206p6,1
ENSDARG00000070590	17	43547312	43567956	-	nt5c1ab
ENSDARG00000021242	3	15558619	15584730	-	mvp
ENSDARG00000074548	23	24311497	24326681	-	zbtb17
ENSDARG00000104365	2	31800954	31802920	+	si:dkeyp-13d12,2
ENSDARG00000031588	12	38755162	38800368	+	si:dkey-239b22,1
ENSDARG00000070826	25	20021812	20029433	+	bpgm
ENSDARG00000077176	16	13964919	13969044	+	zgc:174888
ENSDARG00000018984	6	55925603	56013158	-	eya2
ENSDARG00000020656	13	40689122	40833599	+	prkg1a
ENSDARG00000002656	20	31843070	32003089	-	stxbp5a
ENSDARG00000076919	7	31357396	31488472	-	igdcc4
ENSDARG00000025089	13	40056597	40112923	-	loxl4
ENSDARG00000090986	14	8332194	8395765	+	CENPF
ENSDARG00000009870	12	2470786	2487770	-	mapk8b
ENSDARG00000016527	6	58575577	58621300	+	helz2
ENSDARG00000069471	1	46449556	46460558	-	mhc1zca
ENSDARG00000070387	25	36711529	36713811	-	nudt7

ENSDARG00000043238	15	5483541	5575763	-	atg16l2
ENSDARG00000101178	14	23631267	23672802	+	SH3RF2
ENSDARG00000009040	3	17874235	17881198	-	si:ch73-141c7,1
ENSDARG00000059252	17	25309960	25313056	-	zpcx
ENSDARG00000087575	3	24521885	24530232	+	URGCP-MRPS24
ENSDARG00000074677	1	34882062	34921533	-	frem3
ENSDARG00000062325	25	14069047	14269677	+	shank2
ENSDARG00000100280	6	57401437	57434665	-	znfx1
ENSDARG00000061746	5	21435933	21497253	-	arhgef9a
ENSDARG00000009123	20	34066551	34074289	-	sele
ENSDARG00000005749	22	31096349	31110475	-	cand2
ENSDARG00000073699	11	11291319	11317662	-	col9a1a
ENSDARG00000043249	21	45674662	45800677	-	irf1b
ENSDARG00000045947	12	4340966	4444705	-	hrc
ENSDARG00000101471	18	12168574	12260806	+	fgd4a
ENSDARG00000104139	16	10885786	10930006	+	atp1a3b
ENSDARG00000076411	3	16826295	16862540	+	ptrfa
ENSDARG00000102126	8	9072487	9072605	+	5S_rRNA
ENSDARG00000102987	16	36159258	36188051	-	capn7
ENSDARG00000086746	21	5781884	5816502	+	si:ch73-209e20,3
ENSDARG00000027797	23	26870469	26961676	-	adcy6b
ENSDARG00000074829	16	12626633	12669820	+	rasip1
ENSDARG00000074079	9	37136545	37149379	+	si:dkey-3d4,3
ENSDARG00000008030	6	1641806	1664198	+	myl9b
ENSDARG00000076529	10	4095573	4116145	+	mn1a
ENSDARG00000077054	14	35536562	35544041	-	fam198b
ENSDARG00000100870	9	51243130	51251502	-	CABZ01053748,1
ENSDARG00000013415	16	30562910	30606392	-	lmna
ENSDARG00000006598	16	26656984	26663672	-	sgk2b

ENSDARG00000103542	16	4458253	4488590	-	vegfaa
ENSDARG00000040777	9	37157499	37165084	+	inhbb
ENSDARG00000075282	9	8450909	8475541	-	irs2b
ENSDARG00000012674	11	17830054	17860142	+	rpusd4
ENSDARG00000096562	12	554562	622546	-	wu:fj29h11
ENSDARG00000043821	9	28530363	28588325	-	klf7b
ENSDARG00000079378	15	18012375	18159971	-	phldb1b
ENSDARG00000011571	6	11445584	11469253	+	calcrlb
ENSDARG00000102416	16	36395410	36400391	+	CR974440,1
ENSDARG00000073911	14	33096823	33114362	-	tmem255a
ENSDARG00000016439	10	24476246	24524019	+	slc7a1
ENSDARG00000031702	12	6325145	6418106	+	prkg1b
ENSDARG00000079900	7	24458401	24541867	+	shroom4
ENSDARG00000092166	23	16962809	16991848	+	si:dkey-147f3,8
ENSDARG00000091293	18	39688247	39797228	-	dmxl2
ENSDARG00000088699	17	23800426	23875002	-	pdzd8
ENSDARG00000004115	14	16178273	16448609	-	mgat4b
ENSDARG00000103551	12	36193087	36242090	-	rnf213b
ENSDARG00000055678	20	26509747	26568217	-	akap12b
ENSDARG00000042210	18	9354098	9470930	+	sema3ab
ENSDARG00000099717	21	2066071	2075568	+	si:rp71-1h20,9

Supplementary table 5. Gene list related to the heatmap depicted in Fig. 7 panel A.

kel

atf5a

klf1

ASTE1

epor

nmt1b

tal1

hif1al2

si:ch211-262i1.3

si:dkey-106c17.3

shag

SLC29A1

plagl2

tb celb

col 18a1

sema3b

chl1a

gata1a

si:ch211-227m13.1

si:ch211-117i20.2

sh3bp5b

add2

sh3bp5b

atxn1b

rhd

gp1bb

ggact.2

cyp4v7

slc4a1a

rell2

tubb1

si:dkey-25o16.2

si:ch211-207b24.4

slc16a10

kcnj5

chrm2a

alas2

rbm 38

si:dkey-93h22.7

tmod4

slc25a37

cahz

rfesd

igfbp1a

vtg1

vtg7

vtg5

vtg2

nppb

si:dkey-208m12.2

Supplementary table 6. List of forward and reverse primers.

organism	Gene	Forward 5' - 3'	Reverse 5' - 3'
Dre	dnmt1	GGGCTACCAGTGCACCTTTG	GATGATAGCTCTGCGTCGAGTC
	dnmt3a2	TAGGAAAGGCTTGTTTGAGGG	GCGTGAGATGTCTTTCTTGTC
	dnmt3b2	CGCTACATTGCCTCTGAGA	GCCAGATGTTTCCTAGTGATG
	epoa	TTGCCTTACTGCTGATGGTG	TGACTGGACCTCCTGAGCTT
	hk2	CGGCGTCTGAATAAGATGGT	TGGCGTTGTTGTGTGTTTTT
	hmox1a	CAGCCCATTTACTTCCCTCA	GGACTGCTCTTGCCAATCTC
	ldha	TCACCCGGTTTCTACTTTGG	CTGAACACCCCACAAGGTCT
	p27	GAAGGGCTCCGATATTCACA	CACGATGAGTCGAGACAGGA
	col1a1	CAGACTCCACCTGCTTATTCTACAC	TTGACATCGCCCCTATGGACGTTG
	col1a2	CATGAGGTAGTTTAAACCTTACGG	GGACATTGGCCCAGTCTGTTTCAA
	col15	GATTTCGAGGGTTCTGGTGA	ATACACCTGGCTCACCCTTG
	actc1b	GATCTGGCACCACACCTTCT	CTCATAGACGGGGACGTTGT
	cahz	AAGTATGACCCAGCCACCAC	GTCATCACTGCTTCCCCAAT
	chata	ACAATCCTCAGACGGACCAC	CAGGGAAAGGTATTGGCAGA
	fgf13a	TACATTATGTTGCGGCAGGA	CTTCGTTACGATCCCCTTCA
	gata1a	TTTCACCTCCCCCAAAGAG	TGGACTCAGCATGTGTGGA
	hif1al2	TCTTTGAATGGCAAGGTCATC	CCAACCAGTCAGTTTCGTGTT
	hpda	AGACGGCAAAAATCAAGGTG	AGGACGGTCAGGTTTCCTCT
	kdm6a	GCATGGATGATGAACAGTGG	AGGGGAGGTGATTGTGACTG
	smyhc1	ACCTCATCAAGGGTTTGTGC	CTGTTTGGTGTCCAGGGATT
	tfr1a	ATCAAGCCAGGACAACCATC	GCAGACAGTTTTGGGAGAGC
	tgf2bl	ACAGAGCAGCGAGTGGAAAT	TACAGCAGGGACAGTGAACG
	wnt2ba	GATGAAGACGGGGACTTTGA	CTGGCATCCTTGACCATTCT
	atf2	CAAGCATGAGATGACCCTGA	AGTCCAGCGGCAACTTTTTA
	atf6	TGATGTCTTCATTGCCATTCA	TGGTTTCTCTCCATCCATCTG
	c-Jun	CCACAAGGCTCTGAAACACA	CACAGAAACTGGGTCGGAGT
	creb1a	GTTCCCAGCAATCAAGTGGT	CCTCCCTGTTCTTCATCAGC
	creb1b	TCCAGGAGTTCCAAGAATCG	TGGTCATGGTGAGAGTCTGC
	elk1	GGGTCTTCGCAAGAACAAAA	GGTTTGGTCTGGATGGACAG

	mef2ca	CACGGACATGGACAAAGTTCT	TTAGACTGAGGGATGGCACAC
	mef2cb	TGCTGTGCCCTCTTCTACCT	AGTTGCGGTGGTTTCCATAG
	gapdh	GTGGAGTCTACTGGTGTCTTC	GTGCAGGAGGCATTGCTTACA
Nfu	dnmt1	GTCTCTGCCAGACGATGTCA	GCAAGGCTTTCACCTTTGAG
	dnmt3a	ATTTCTTCGCCAACAACCAC	GCCCACAGTGATGGAATCTT
	dnmt3b	CATGAGAAGGGTGGAGTGGT	AGCTGCCAACTCGATGATCT
	col1a1	GACCCACAGGATCTCAAGGA	ACCACTATCACCAGCGGTTC
	col1a2	CAAGAACAGCGTTGCCTACA	CAATGTCCAAAGGTGCAATG
	col11a1	ATGGAGATGCAGAGGTGGTC	AATCCCGATGTTCTCGTCAC
	col15a1	TACCATGCCTTTTCCAGACC	TTCTCCTCGTTCCCCTTTTT
	ir	TGCCTCTTCAAACCCTGAGT	AGGATGGCGATCTTATCACG
Hsa	dnmt1	ATCCGAGGAGGGCTACCTG3	CACTTCCCGGTTGTAAGCAT
	dnmt3a	AGCCCAAGGTCAAGGAGATT3	GTTCTTGCAGTTTTGGCACA
	dnmt3b	TCAGGATGGGAAGGAGTTTG	CTGCAGAGACCTCGGAGAAC
	col1a1	GAACGCGTGTCATCCCTTGT	GAACGAGGTAGTCTTTCAGCAACA3
	col3a1	AACACGCAAGGCTGTGAGACT3	GAACGAGGTAGTCTTTCAGCAACA
	col11	GGTCTGCAGTCGCAATTTCG	CCACCTAGAGGACCACGGCT
	col15	AGGCCCTGGATGCACAATG	GACCAATTGCCGTTGG
	pri-miR-29ab	TGAATGTGTGAGTTCTTGGAAGT	GATCCCCAAGCGCAGGACT
	pri-miR-29bc	AGATTTGCATTGACGTCCGG	CCAGGCTAAGGATCAGCTCA
	p0	TCGACAATGGCAGCATCTAC	ATCCGTCTCCACAGACAAGG
